# Introducing Alternative-Based Thresholding for Defining Functional Regions of Interest in fMRI

**DOI:** 10.3389/fnins.2017.00222

**Published:** 2017-04-21

**Authors:** Jasper Degryse, Ruth Seurinck, Joke Durnez, Javier Gonzalez-Castillo, Peter A. Bandettini, Beatrijs Moerkerke

**Affiliations:** ^1^Department of Data-Analysis, Ghent UniversityGent, Belgium; ^2^Department of Psychology, Stanford UniversityPalo Alto, CA, USA; ^3^Section on Functional Imaging Methods, Laboratory of Brain Cognition, National Institute of Mental Health, National Institutes of HealthBethesda, MD, USA

**Keywords:** fMRI, functional ROI, localizer task, effect size, alternative distribution

## Abstract

In fMRI research, one often aims to examine activation in specific functional regions of interest (fROIs). Current statistical methods tend to localize fROIs inconsistently, focusing on avoiding detection of false activation. Not missing true activation is however equally important in this context. In this study, we explored the potential of an alternative-based thresholding (ABT) procedure, where evidence against the null hypothesis of no effect and evidence against a prespecified alternative hypothesis is measured to control both false positives and false negatives directly. The procedure was validated in the context of localizer tasks on simulated brain images and using a real data set of 100 runs per subject. Voxels categorized as active with ABT can be confidently included in the definition of the fROI, while inactive voxels can be confidently excluded. Additionally, the ABT method complements classic null hypothesis significance testing with valuable information by making a distinction between voxels that show evidence against both the null and alternative and voxels for which the alternative hypothesis cannot be rejected despite lack of evidence against the null.

## 1. Introduction

Functional magnetic resonance imaging (fMRI) is an important technique to unravel the working of the human brain in cognitive, clinical and psychological research. To identify task-related areas, a statistical test is performed in each voxel, which induces a huge multiple testing problem as over 100,000 voxels are tested simultaneously, inflating the false positive rate. To account for this, thresholding is typically made very conservative. Such conservatism boosts the ability to exclude activation when there is none (specificity) but dramatically reduces the ability or power to detect activation when it is present (sensitivity).

As a means to counter the multiple testing problem in fMRI, a subsection of the brain, called a region of interest (ROI), is the target of the research hypothesis in progressively more studies. The statistical advantage of an ROI analysis lies in the reduction of the number of voxels to be tested and, as a consequence, the reduction of the impact of the multiple testing problem which leads to less stringent procedures and more sensitive analyses (Poldrack, 2007). In order to define an ROI, researchers can use macro-anatomic landmarks such as gyri and sulci, but this does not necessarily lead to a functionally homogeneous ROI (Uematsu et al., 1992; Amunts et al., 2000; Farrell et al., 2007). Instead, researchers can use information from an independent localizer task, leading to so-called functional ROIs (fROIs). This localizer task is chosen based on previous research where a specific comparison of stimuli or tasks has shown activation of the ROI, e.g., processing faces vs. houses to define the fusiform face area (Kanwisher et al., 1997; McCarthy et al., 1997). The fROI tradition was first applied to define regions in the visual cortex (Malach et al., 1995; Tootell et al., 1995, 1998) and subsequently found its way into other research fields (Kanwisher et al., 1997; McCarthy et al., 1997; Epstein and Kanwisher, 1998; Downing et al., 2001).

Despite some debate on functional localization in fMRI (Friston et al., 2006), the identification of fROIs has become increasingly important (Saxe et al., 2006; Duncan et al., 2009; Duncan and Devlin, 2011), mainly because of the advantages fROIs provide over anatomically based ROIs. First of all, fROIs improve the sensitivity of analyses over individuals (Nieto-Castañón et al., 2003; Saxe et al., 2006; Nieto-Castañón and Fedorenko, 2012). The location and extent of brain regions that respond to specific stimuli or tasks may differ substantially across individuals (Duncan et al., 2009), leading to a loss of sensitivity when combining statistical parametric maps (SPMs) across subjects. The fROI method deals with this inter-subject variability by defining an fROI in each individual subject, summarizing the signal within this region and testing for activation on the group-level using the fROI aggregates of all participants. Secondly, the identification of fROIs allows addressing additional research questions about a specific brain region. For example does visual mental imagery rely on the same functional areas as visual perception (Seurinck et al., 2011)? Finally, the signal in an fROI can serve as input for biological markers (e.g., a measure of clinical depression).

Once the fROI is defined, the behavior of the region as a whole is examined in the primary experimental task by summarizing the signal across the voxels of the fROI during this main experimental manipulation. This approach is optimal since the localizer is independent of the data analyzed in the primary experiment (Poldrack et al., 2011). To avoid potential bias in the results of the primary experiment, the fROI needs to be as spatially accurate as possible. If the fROI is less specific and includes voxels that are incorrectly labeled as active during the localizer task, i.e., false positives (FPs), noise voxels are introduced in the subsequent analyses. Similarly, if the fROI is less sensitive and excludes voxels that are truly involved in the localizer task, i.e., false negatives (FNs), relevant information from signal voxels is missing in further analyses. Spatial accuracy of (f)ROIs is not only extremely important in the context mentioned here, but also in the context of functional and effective connectivity. Smith et al. (2011) showed that inaccurately defined ROIs, which take the role of nodes in a network, severely bias the network analysis. Currently, two common methods are used to define an fROI: (a) researchers draw a fixed geometric shape (e.g., a sphere) around the peak voxel near the anatomical region they are interested in (Miller and D'Esposito, 2005; Blankenburg et al., 2006; Aleong and Paus, 2010; Tibber et al., 2010; Kühn et al., 2011), or (b) researchers define the fROI as a cluster of active voxels detected in the proximity of that same anatomical region (Kanwisher et al., 1999; Grill-Spector et al., 2004; Spiridon et al., 2006; Morris et al., 2008; Yovel et al., 2008; Axelrod and Yovel, 2010). Previous research has demonstrated that the former method is suboptimal with respect to spatial accuracy of the fROI (Duncan et al., 2009; Berman et al., 2010; Duncan and Devlin, 2011).

To detect clusters of active voxels that overlap with specific anatomical regions, null hypothesis significance testing (NHST) procedures for voxelwise testing are typically used. However, this might be an inapt strategy in the context of functional localization. Even in smaller anatomical regions of the brain, a sole focus on pursuing specificity and controlling the FP rate through stringent thresholding can dramatically increase the FN rate and reduce the sensitivity (Lieberman and Cunningham, 2009). Duncan and Devlin (2011) suggested that conservative statistical thresholds are unreliable to identify fROIs with the typical small amount of data collected for localizers. While it is advised to use more lenient thresholds when defining fROIs, there is no theoretical basis to adjust thresholding in terms of controlling a particular FP rate, potentially provoking an *ad hoc* adjustment of significance levels. Furthermore, lenient thresholding provides a partial solution as sensitivity is only increased by allowing more FPs and not through direct control of FNs.

In this paper, we study the potential of a procedure introduced by Durnez et al. (2013) which was originally developed in the context of pre-surgical fMRI. Pre-surgical fMRI studies guide the resection of brain lesions such as tumors to preserve vital brain tissue. FNs in this context can have dramatic consequences as brain regions involved in specific functions may be removed. Durnez et al. (2013) therefore present a method that incorporates information on both FPs and FNs. Since the presence of both FPs and FNs compromises the spatial accuracy in defining fROIs, a formal approach with a stronger focus preventing FNs is also needed in this context. The procedure requires specification of an alternative of interest which enables the construction of valid tests directly aimed at the detection of effect sizes of interest (Rouder et al., 2016).

In NHST, the classical *p*-value measures evidence against the null hypothesis. Thresholding this *p*-value at a given level α enables to control the FP rate. The alternative-based thresholding method (ABT) as described by Durnez et al. (2013) presents a symmetrical, alternative *p*-value measuring evidence against the alternative hypothesis that the change in signal is of a particular magnitude (Moerkerke et al., 2006). As a result, the FN rate can be controlled directly by thresholding these *p*-values at level value β. Thresholding both *p*-values results in a layered statistical parametric map (LSPM) of the brain. The *active layer* contains voxels which show strong evidence against the null and lack of evidence against activation during the localizer task. The *inactive layer* consists of voxels that exhibit strong evidence against true activation and no evidence against the null. The *practically insignificant layer* represents voxels with strong evidence against the null hypothesis but also against activation. The *uncertainty layer* consists of voxels that show lack of evidence against both the null and the alternative. Hence, while the null cannot be rejected for these voxels, true activation cannot be confidently excluded.

In this paper, we study the potential of ABT in the context of defining fROIs. Using simulated data and real data, we assess the information that is captured in the different layers of the LSPM and investigated whether the method can effectively distinguish between scientifically relevant and scientifically irrelevant voxels. In the latter voxels, the task-related signal change is too small to be considered of scientific interest. In both settings (simulated and real data), we evaluate the influence of different parameters of the data analysis procedure on the prevalence and content of each of the layers. Simulations additionally allow to study the influence of parameters inherent to the localizer task itself.

This paper is organized as follows. In Section 2, the ABT method for defining fROIs and the material and procedures to study its results are discussed. Results are shown in Section 3, followed by a discussion in Section 4.

## 2. Materials and methods

### 2.1. Alternative-based thresholding for defining fROIs

In voxelwise fMRI data analysis, typically a General Linear Model (GLM) is fitted in each voxel to regress the measured times series of the BOLD signal onto the design of the experimental task. For each voxel, a test statistic *T* is obtained of the form $\hat{\Delta }/\text{SE}\left(\hat{\Delta }\right)$ with $\hat{\Delta }$ an unbiased estimator for the true effect magnitude of activation Δ and $\text{SE}\left(\hat{\Delta }\right)$ its corresponding standard error.

Assuming the distribution of *T* is known (e.g., Gaussian) when there is no task-related activation (null hypothesis *H*_0_:Δ = 0 is true), the classical *p*-value for a one-sided test in a specific voxel equals *P*(*T* ≥ *t*|*H*_0_) with *t* the observed test statistic for the voxel. We further denote this *p*-value by *p*_0_. The smaller *p*_0_, the more evidence against *H*_0_. Thresholding *p*_0_ against α enables direct control of the FP rate at level α. This α value can be defined based on uncorrected thresholding (e.g., α could simply equal 0.05 or 0.001 for each statistical test) or multiple comparisons corrections such as false discovery rate (e.g., *q* value = 0.05, which leads to a specific threshold for each individual subject) (Benjamini and Hochberg, 1995; Genovese et al., 2002; Efron, 2004) or familywise error rate using random field theory (Adler, 1981; Friston et al., 1991; Nichols and Hayasaka, 2003). For the remainder of this paper, the term NHST concerns statistical testing that considers a threshold α to control the FP rate, regardless of how this value is defined. Given the α value, two outcomes are possible for each voxel: either its *p*_0_-value ends up below the α-threshold, leading to a rejection of *H*_0_ and the conclusion that the voxel was active during the task, or it does not.

ABT extends this procedure by contrasting the null of no activation with an alternative hypothesis *H*_1_ that states that the effect magnitude of the underlying activation Δ equals Δ_1_ ≠ 0 (and > 0 for a one-sided test), the effect magnitude expected when true activation is present. This magnitude should be large enough to reflect a scientifically relevant effect in the specific context of the experiment in question. For power calculations for fMRI, guidelines for choosing a meaningful effect magnitude are available (Desmond and Glover, 2002; Hayasaka et al., 2007; Mumford and Nichols, 2008). Alternatively, researchers can estimate this effect magnitude on the basis of (localizer) data of previous subjects or studies. Note that in the literature, the term “effect size” may refer to a standardized or an unstandardized measure of effect. For ABT, we use the term for an unstandardized effect: it is the effect magnitude on the scale in which the signal is modeled (e.g., % BOLD signal change).

In each voxel, ABT complements *p*_0_ with an alternative *p*-value *p*_1_ = *P*(*T* ≤ *t*|*H*_1_) which measures evidence against *H*_1_ in the direction of *H*_0_ (Moerkerke et al., 2006; Durnez et al., 2013). The smaller *p*_1_, the more evidence against true activation. To obtain this *p*-value, the distribution of *T* under *H*_1_ needs to be known. Durnez et al. (2013) do not consider Δ_1_ as one fixed value but as a distribution of possible true underlying effects with an a priori defined expected value, μ_Δ_1__ to account for variability of effect sizes (ESs) across voxels, brain regions, subjects etc. Durnez et al. (2013) pose that the test statistic for a given voxel *i* has the following distribution under the alternative hypothesis:

(1) $$\begin{array}{r} T_{i}\sim N\left(\frac{\mu_{\Delta 1}}{SE\left({\hat{\Delta }}_{i}\right)},\frac{SE{\left({\hat{\Delta }}_{i}\right)}^{2}+\tau^{2}}{SE{\left({\hat{\Delta }}_{i}\right)}^{2}}\right), \end{array}$$

where τ represents the standard deviation of Gaussian variation on ESs under task-related activation across voxels. τ also has to be defined a priori. The values of μ_Δ1_ and τ are fixed for the entire brain, based on prior knowledge, while SE(${\hat{\Delta }}_{i}$) is not.

**Figure 1:**
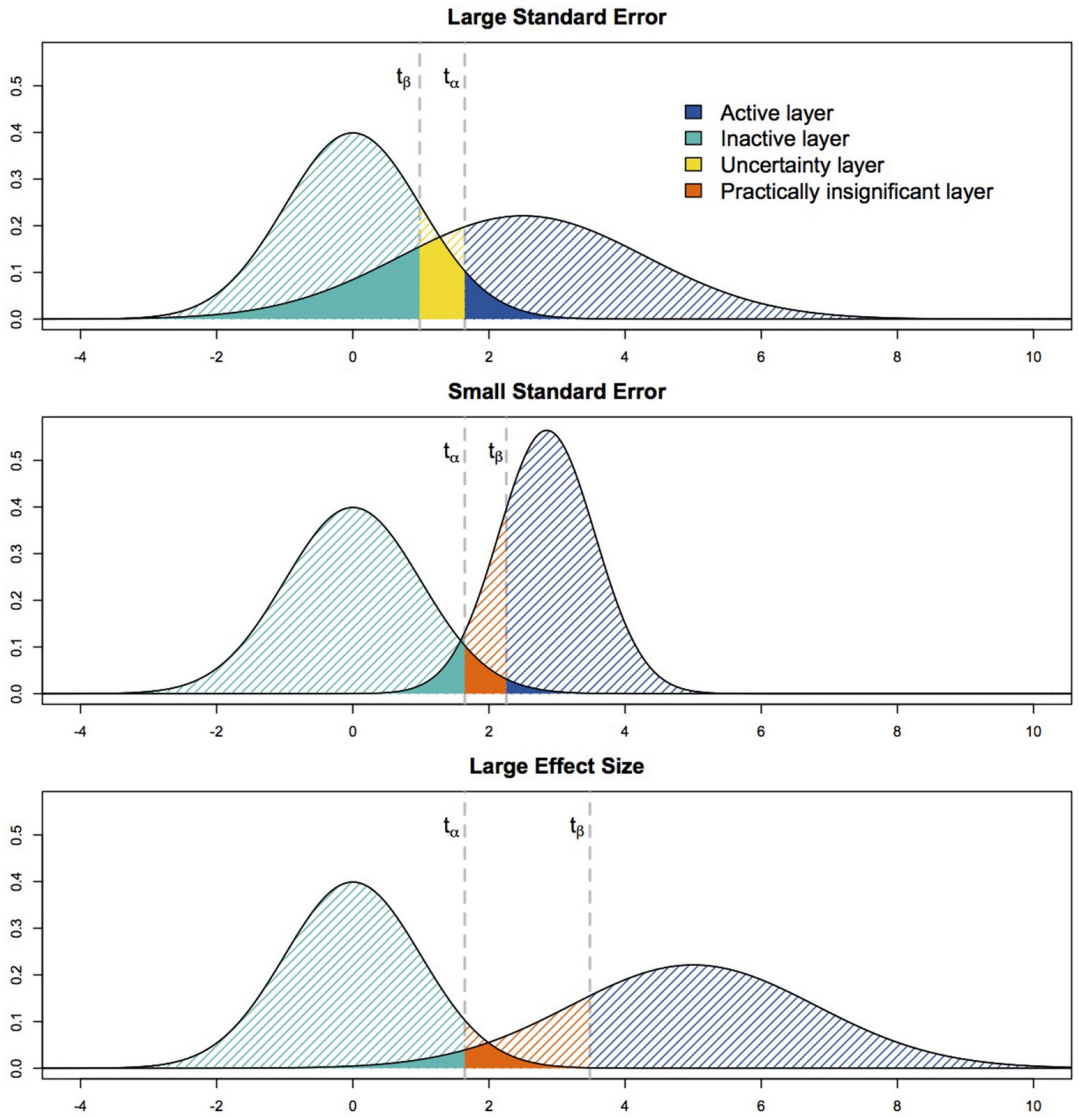
**The different scenarios for each voxel when thresholding ***p***_**0**_ and ***p***_**1**_**.

As with NHST, the *p*_0_-value is thresholded against α. This α threshold can again be defined based on uncorrected thresholding or can be obtained through multiple comparisons corrections. In parallel to α and the FP rate, β represents the probability that a FN occurs if the experiment would be repeated an infinite number of times. Thresholding *p*_1_ against β enables control of the FN rate. If *p*_1_ > β, there is no sufficient evidence to reject *H*_1_ (true activation of magnitude Δ_1_). If *p*_1_ < β, there is enough evidence against *H*_1_ to reject it. In ABT, binary decisions can be made for both *p*_0_ and *p*_1_, resulting in four possible outcomes. As a consequence, the SPM now contains four different kind of layers (see Figure 1), resulting in a layered statistical parametric map (LSPM). The first layer, the active layer, contains the active voxels of which both types of evidence point at the presence of true task-related activity (*p*_0_ < α, *p*_1_ > β). The second, or inactive, layer contains the voxels for which we can confidently conclude absence of activity during the experiment (*p*_0_ > α, *p*_1_ < β). The third layer, called uncertainty layer, contains the voxels of which we cannot confidently state presence or absence of true activity, because both *H*_0_ and *H*_1_ cannot be rejected (*p*_0_ > α, *p*_1_ > β). Finally, the fourth layer contains practically insignificant voxels or voxels with an effect that is (1) statistically significant with respect to the null hypothesis of no effect, but (2) at the same time too small for evidence in favor of an alternative hypothesis with an ES of magnitude Δ_1_ (*p*_0_ < α, *p*_1_ < β).

ABT univariately categorizes each voxel in a layer of the LSPM depending on its test statistic *t*. However, for each voxel only three of the four layers are possible, according to whether the cut-off value under *H*_0_, *t*_α_, is larger or smaller than the cut-off value under *H*_1_, *t*_β_ for that specific voxel (see Figure 1). Depending on which of these situations arises, a voxel could either be categorized as active, inactive or uncertain (*t*_α_ > *t*_β_) or active, inactive or practically insignificant (*t*_α_ < *t*_β_). Both parameters inherent to the scanning characteristics during the localizer experiment itself as well as parameters defined during the data analysis influence the mutual location of these cut-off values.

First, as shown in Equation (1), the standard error (SE) of the estimator ${\hat{\Delta }}_{i}$ of a specific voxel has an impact on the shape of the alternative distribution, since it affects its variance parameter. As the SE increases (for a constant α and β), the alternative distribution expands, which shifts the cut-off value under *H*_1_ to the left (see Figure 1, upper panel). This leads to the scenario where the uncertainty layer becomes a possibility for that voxel. As the SE decreases, the cut-off value under *H*_1_ shifts to the right, leading to the emergence of the practically insignificant layer (see Figure 1, middle panel). The SE of a voxel is largely dependent on the characteristics of the localizer task used to define the fROI and the scanning circumstances. First, the SE will decrease when the amount of noise in the localizer data decreases. Noise sources could be thermal noise from the scanner, motion noise, physiological noise, non-task-related noise such as spontaneous neural activity, etc. Secondly, the SE decreases as the number of scans increases during the localizer task. Finally, the SE decreases when the variance in the design matrix of the experiment, or the model, increases. Factors that influence the variance of the design include the type of design (blocked designs are accompanied with more variance than event-related designs), whether null events were added, whether ceiling or bottom effects in the design are avoided, what hemodynamic response was convoluted with the model, etc. It is important to note that both the number of scans and the variance of the design typically do not vary across voxels. The amount of noise, however, can vary at voxel level.

Next to the SE, parameters that have to be defined during the data analysis also influence whether *t*_α_ > *t*_β_ or *t*_α_ < *t*_β_. First, *t*_α_ increases with a decreasing (more stringent) α. Symmetrically, choosing a β value which represents more importance to avoiding FNs, i.e., a smaller value, shifts *t*_β_ to the left. Secondly, as the mean of the alternative distribution, μ_Δ1_, increases, so does *t*_β_, leading to more practically insignificant voxels (see Figure 1, bottom panel). Finally, τ (standard deviation of ESs over voxels) influences the variance of the ES under the alternative, similarly as the voxelwise SEs. In summary, we expect the number of voxels in the uncertainty layer to increase as one of the following parameters varies while the others remain constant: μ_Δ1_, β, the number of scans or the design variance decrease or α, τ or the amount of noise present in the data increase. The number of practically insignificant voxels will increase when the opposite is true.

For the definition of fROIs, ABT may provide valuable information in addition to NHST. While directly controlling both FPs and FNs at prespecified levels (α and β), it provides areas of voxels of which we can reliably say that they are part of the fROI (active layer) as well as areas of voxels which we can safely exclude (inactive layer). Additionally, it provides areas in between, either with voxels that have an effect that is practically insignificant or with voxels for which both hypotheses cannot be rejected (uncertainty layer). Classical NHST coincides with selecting voxels that lie in either the active or the practically insignificant layer.

The goal of this study is to (1) study the influence of the different parameters and data characteristics as described above using simulated and real data and, (2) to examine in which situations the ABT has an added value when defining an fROI.

### 2.2. Study 1: simulations

The two parameters that created the simulation conditions were the number of time points of the experimental design, which could be 50, 100, or 150 scans (time points), and the amount of Gaussian noise added at all time points (σ_*noise*_ was either 3, 5.5, or 7% BOLD signal change). While 5.5 and 7 are realistic noise values for fMRI data, a σ_*noise*_ value of 3 represents a rather extreme situation enabling evaluation of the information that is present in the practically insignificant layer (emerges when SE is small). The combination of these two variables resulted in nine simulation conditions. In each condition, a set of 500 4D images (time points × X × Y × Z with X = Y = 64 and Z = 40) were generated using functions of the R package neuRosim (Welvaert et al., 2011).

We used a blocked design in which blocks of the active condition were altered by blocks of non-activity. Each block had a duration of 25 s. The number of blocks was dependent on the number of scans in each of the nine conditions. The TR was 2 s. In two spheres of 515 voxels at realistic coordinates for the bilateral hippocampi in MNI space, signal was added in half of the time points to simulate subjects only responding during the subset of the task blocks. These spheres represent activated regions. The location of activation does not affect the simulation results, but we wanted to simulate data typical for a localizer task. Within each image, the ES varied between the two spheres. In one sphere, the voxels had an effect size of 1% and in the other an effect size of 2% BOLD signal change. Since we adhered to the definition of contrast to noise ratio (CNR) as the ES divided by σ_*noise*_, the parameter choices resulted in a range of CNR values going from 0.14 to 0.67. Finally, each scan within the 4D images was spatially smoothed using a 3D Gaussian kernel with σ = 3.40. This induced larger spatial correlation between neighboring voxels and smaller spatial correlation between voxels that are located further away from each other. There was no temporal correlation included in the simulations.

A GLM was fitted to the timeseries in the 4D image, with its predictor being the blocked design matrix described above convolved with a double-gamma hemodynamic response function. This resulted in a *T*-statistic map. Classical *p*_0_-values were obtained using the null distribution of these *T*-statistics, a *T*-distribution (degrees of freedom: number of time points - 2). Alternative *p*_1_-values for each voxel were obtained using the distribution of the *T*-statistics under the alternative distribution defined by μ_Δ_1__ and τ. As input for ABT, we set μ_Δ_1__ equal to 1.5% BOLD signal change. This implies that in each image, one region had a scientifically relevant ES (2%) while the ES in the other region (1%) was smaller and not considered to be of scientific interest. Based on these ESs, τ was set at 0.5% BOLD signal change. α was either 0.05 or 0.001, while β was either 0.1, 0.2, or 0.3.

To examine the information obtained through ABT, the number of scientifically irrelevant (ES = 1% BOLD signal change) and scientifically relevant (ES = 2% BOLD signal change) signal voxels as well as the number of noise voxels were computed in each layer. This was done for each simulated image and average numbers over all images within simulation conditions were calculated.

### 2.3. Study 2: real data example

We used preprocessed data of one subject from previously published research where three subjects were engaged on a visual stimulation combined with a letter/number discrimination task (Gonzalez-Castillo et al., 2012). Each subject completed 100 runs of 170 timepoints, in which an ON/OFF block paradigm was used. Data were preprocessed using AFNI where the first five scans in each run were discarded, effects of physiological noise and slow blood-oxygenation level fluctuations were removed, slice-time correction and motion correction was performed and within-subject interrun spatial coregistration was executed. For more information on the specifics of the data acquisition and preprocessing, see the Supporting Information accompanied with Gonzalez-Castillo et al. (2012).

**Figure 2:**
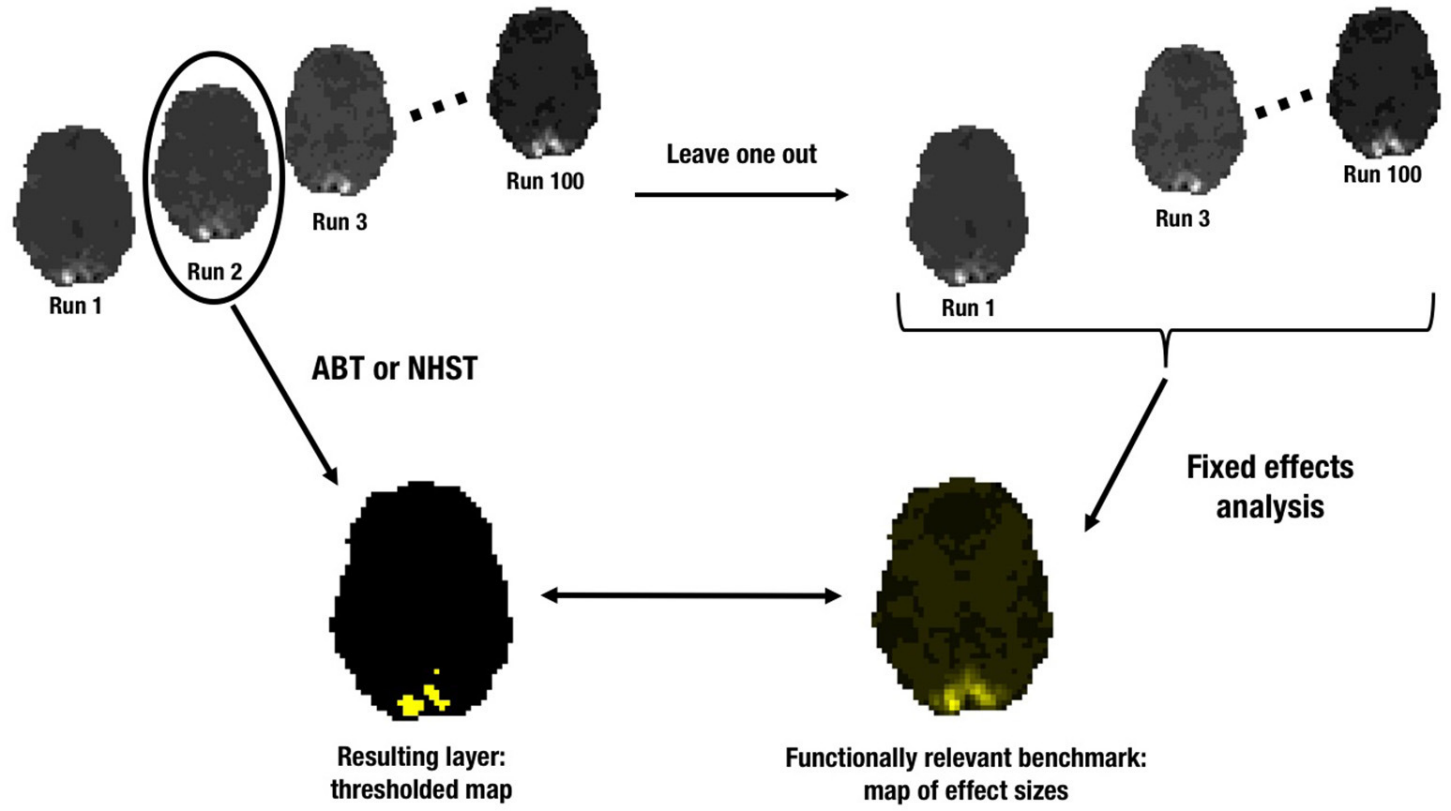
**Illustration of the leave-one-out cross-validation procedure**.

In order to evaluate the information captured in the four layers of the LSPM, we performed a leave-one-out cross-validation with 100 steps (see Figure 2). In each step, the analysis of one run was evaluated with respect to a reference image or ground truth of underlying ESs using the remaining 99 runs, since a single run is typical for functional localizers. The reference image was constructed as follows. First we computed the contrast estimates for each voxel in each of the 99 runs using the GLM. Next, these 99 parameter estimate maps were combined through a fixed effects analysis resulting in a SPM with an average ES for each voxel across 99 runs. All analyses were performed using FSL^1^ (RRID:birnlex_2067). The SPMs contributing to the reference image were not thresholded before contributing to the fixed effects analysis. The reference image consists of the effect sizes over all voxels.

In the original paper (Gonzalez-Castillo et al., 2012), the authors showed that even simple tasks activate more than 95% of the brain and not only areas that are primarily related to the task. Here, however, we used their data for illustratory purposes, since it permits to examine which information is present in each of the layers in respect of a prespecified effect size in the context of a sparse amount of data, as is the case with functional localization. Our focus is not the detection of whole-brain activation but only those voxels that are part of a specific fROI that exhibits a well-defined amount of activation (quantified through μ_Δ_1__) in the context of a sparse amount of data with a high level of noise.

The single run was analyzed using the ABT method and traditional NHST, both uncorrected and with FDR-corrected thresholding (Efron, 2004). ABT was performed using the same α (0.001 or 0.05) and β (0.1, 0.2, or 0.3) values as defined in the simulation study. Additionally, the threshold for the null distribution of the ABT method was also defined using FDR control with *q* either equal to 0.01 or to 0.05. For uncorrected NHST, α was set at 0.05 or 0.001 as well. For the FDR-corrected NHST, *q* equaled 0.01 or 0.05. To avoid circularity, parameters for the alternative distribution were based on findings as reported in Durnez et al. (2013), i.e., τ = 0.21% BOLD signal change and μ_Δ_1__ either 0.25, 0.50, or 0.75% BOLD signal change. The variation in the choice of μ_Δ_1__ allows to evaluate the performance of ABT for different alternatives. However, we stress that this is done for illustratory purposes only. In ABT, μ_Δ_1__ is a constant parameter that represents from which magnitude on an effect is scientifically relevant.

**Figure 3:**
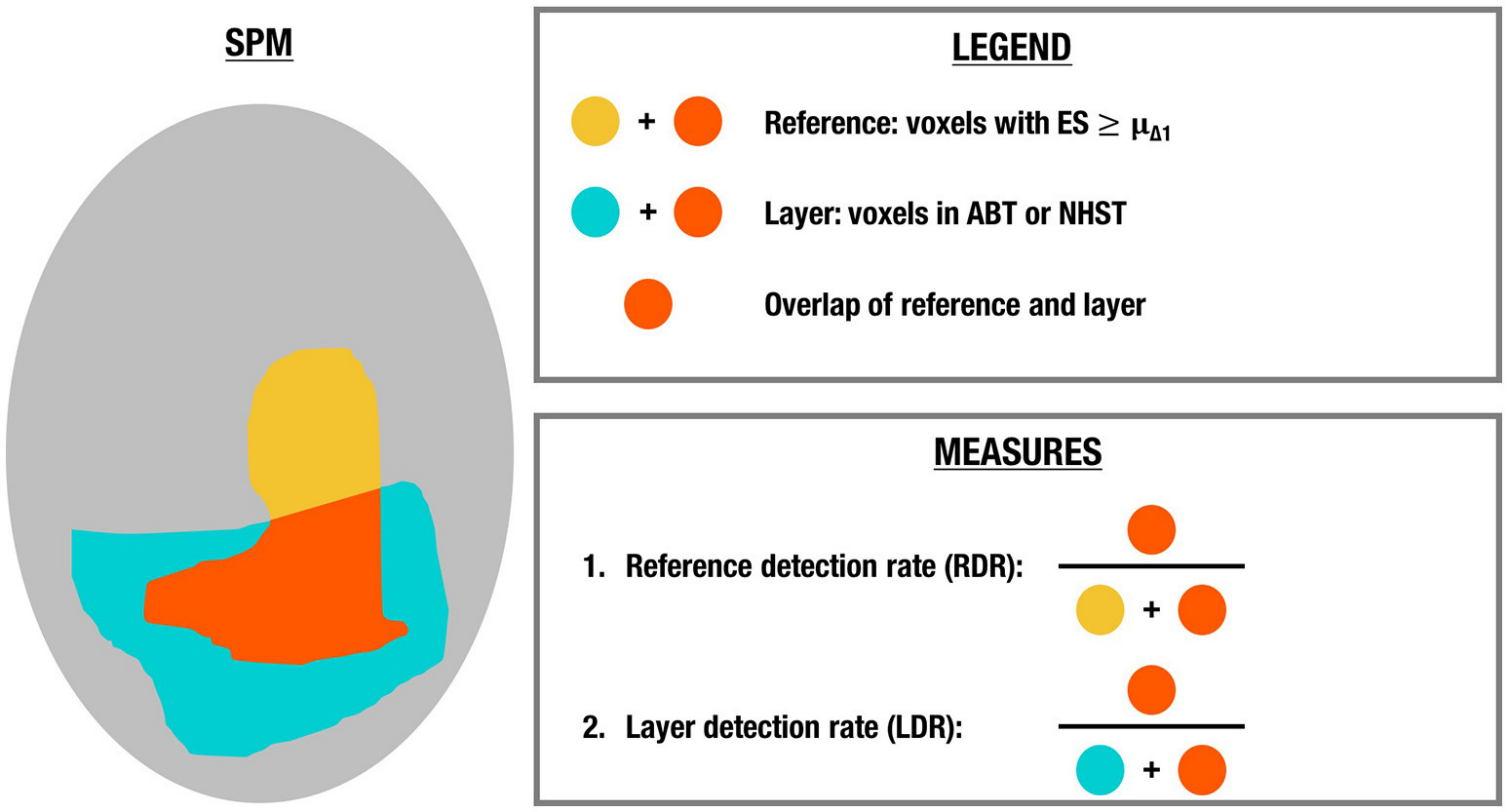
**Illustration of the outcome measures in the real data example**.

Analogous to the simulations, the number of voxels in the layer with a scientifically relevant ES in the reference image, i.e., with an ES larger than or equal to μ_Δ_1__, was evaluated in the active, uncertainty and practically insignificant layer in the LSPM after ABT and in the significant layer after (uncorrected and FDR-corrected) NHST. More specifically, we calculated the reference detection rate (RDR), which is the number of voxels that have an ES ≥ μ_Δ_1__ in the reference map and are part of a certain layer divided by the total number of voxels in the reference map of which the ES ≥ μ_Δ_1__. Additionally, we computed the layer detection rate (LDR), which is the number of voxels that have an ES ≥ μ_Δ_1__ in the reference map and are part of a certain layer divided by the total number of voxels in that layer. These measures were computed in each step and averages were calculated over all runs. See Figure 3 for a visual presentation of these outcome measures. For the inactive layer of the LSPM after ABT and the non-significant layer after NHST, the RDR and LDR were computed using the scientifically irrelevant voxels of the reference image, i.e., voxels that have an ES < μ_Δ_1__. All code used for both the simulation study and the real data example can be found on GitHub (https://github.com/jdgryse/ABT_for_localizing_fROIs).

## 3. Results

### 3.1. Study 1: simulations

**Figure 4:**
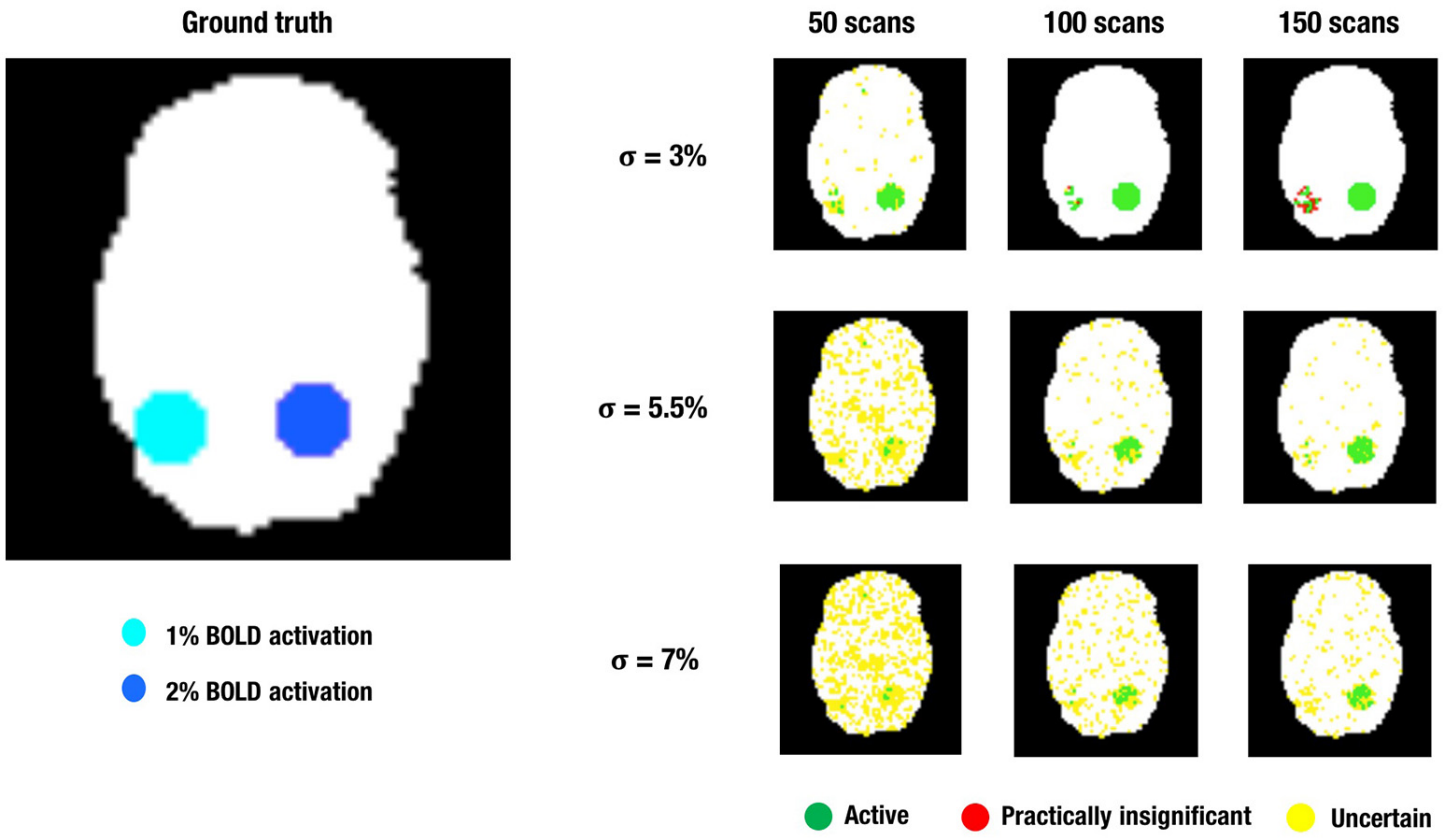
**This figure shows a visual representation of the ground truth (Left)** and the different layers of the LPSM after ABT **(Right)** for one iteration in the simulations. α = 0.001 and β = 0.2 in all scenarios shown.

The results for the different layers are shown in Tables 1–4. The ground truth, active, uncertainty and practically insignificant layer in all nine simulation conditions are shown for one simulation with α = 0.001 and β = 0.2 in Figure 4. Table 5 shows the contribution of the parameters in the simulation design to the total variability in the functionally relevant (ES = 1% BOLD signal change), the functionally irrelevant (ES = 2% BOLD signal change) and the noise (ES = 0% BOLD signal change) voxels for each layer. Effect sizes are based on type II sum of squares. Hence, effect sizes of main effects correspond to those in a model without interactions. We followed the suggestion of Cohen (1988), which stated that effect sizes less than 1% could be interpreted as small, close to 6% could be interpreted as medium, and larger than 14% could be considered large. For the interaction effects, only those with an effect size larger than 6% are discussed in the results below.

**Table 1:** **Average number of voxels for the active layer of the ABT method**.

**σ_*noise*_**	**α**	**β**	**50 scans**			**100 scans**			**150 scans**		
			**ES = 1**	**ES = 2**	**ES = 0**	**ES = 1**	**ES = 2**	**ES = 0**	**ES = 1**	**ES = 2**	**ES = 0**
3	0.05	0.1	302 (17.77)	498 (4.60)	2,181 (59.05)	349 (15.97)	511 (1.64)	379 (21.99)	343 (15.88)	512 (1.24)	60 (8.61)
		0.2	243 (18.85)	487 (6.01)	1,082 (40.93)	194 (16.91)	501 (3.72)	28 (5.52)	170 (16.72)	504 (3.22)	2 (1.39)
		0.3	158 (17.41)	459 (9.28)	342 (21.34)	97 (13.65)	480 (6.17)	3 (1.77)	71 (12.15)	486 (5.30)	0 (0.26)
	0.001	0.1	55 (10.82)	357 (15.66)	45 (7.03)	189 (17.23)	499 (4.02)	34 (5.99)	259 (17.14)	511 (1.66)	27 (5.51)
		0.2	55 (10.82)	357 (15.66)	45 (7.02)	173 (17.03)	497 (4.22)	20 (4.57)	170 (16.72)	504 (3.22)	2 (1.39)
		0.3	55 (10.81)	357 (15.66)	44 (6.87)	97 (13.64)	480 (6.16)	3 (1.76)	71 (12.15)	486 (5.30)	0 (0.26)
5.5	0.05	0.1	138 (16.64)	338 (17.96)	2,246 (59.63)	237 (17.54)	469 (8.13)	2,176 (60.80)	300 (18.15)	498 (4.66)	2,153 (58.25)
		0.2	138 (16.64)	338 (17.96)	2,246 (59.62)	234 (17.56)	469 (8.18)	2,101 (59.34)	248 (18.64)	488 (6.00)	1,184 (42.44)
		0.3	138 (16.64)	338 (17.96)	2,246 (59.54)	186 (17.03)	448 (10.39)	1,199 (42.20)	160 (17.08)	459 (9.14)	370 (22.19)
	0.001	0.1	9 (3.87)	74 (12.56)	45 (7.03)	27 (7.11)	240 (18.80)	34 (60.80)	49 (9.66)	352 (15.89)	30 (5.69)
		0.2	9 (3.87)	74 (12.56)	45 (7.03)	27 (7.11)	240 (18.80)	34 (59.34)	49 (9.66)	352 (15.89)	30 (5.69)
		0.3	9 (3.87)	74 (12.56)	45 (7.03)	27 (7.11)	240 (18.80)	34 (42.20)	49 (9.66)	352 (15.89)	30 (5.69)
7	0.05	0.1	104 (14.63)	253 (19.53)	2,246 (59.63)	171 (17.36)	400 (14.33)	2,176 (60.80)	219 (18.48)	456 (9.73)	2,154 (58.29)
		0.2	104 (14.63)	253 (19.53)	2,246 (59.63)	171 (17.36)	400 (14.33)	2,176 (60.80)	219 (18.50)	456 (9.74)	2,149 (58.41)
		0.3	104 (14.63)	253 (19.53)	2,246 (59.63)	171 (17.36)	400 (14.34)	2,176 (60.28)	192 (18.11)	443 (10.91)	1,578 (49.27)
	0.001	0.1	5 (2.78)	35 (8.24)	45 (7.03)	13 (4.76)	121 (15.52)	34 (5.60)	22 (6.11)	207 (18.16)	30 (5.69)
		0.2	5 (2.78)	35 (8.24)	45 (7.03)	13 (4.76)	121 (15.52)	34 (5.60)	22 (6.11)	207 (18.16)	30 (5.69)
		0.3	5 (2.78)	35 (8.24)	45 (7.03)	13 (4.76)	121 (15.52)	34 (5.60)	22 (6.11)	207 (18.16)	30 (5.69)

**Table 2:** **Average number of voxels for the inactive layer of the ABT method**.

**σ_*noise*_**	**α**	**β**	**50 scans**			**100 scans**			**150 scans**		
			**ES = 1**	**ES = 2**	**ES = 0**	**ES = 1**	**ES = 2**	**ES = 0**	**ES = 1**	**ES = 2**	**ES = 0**
3	0.05	0.1	146 (15.20)	7 (2.99)	40,691 (89.40)	67 (9.75)	0 (0.71)	42,781 (60.80)	28 (5.97)	0 (0.20)	42,803 (58.29)
		0.2	208 (17.70)	15 (4.47)	42,679 (60.42)	67 (9.75)	0 (0.71)	42,781 (60.80)	28 (5.97)	0 (0.20)	42,803 (58.29)
		0.3	210 (17.74)	16 (4.59)	42,711 (59.62)	67 (9.75)	0 (0.71)	42,781 (60.80)	28 (5.97)	0 (0.20)	42,803 (58.29)
	0.001	0.1	148 (15.29)	7 (3.02)	40,756 (89.17)	165 (15.97)	3 (1.64)	44,578 (21.99)	169 (15.66)	2 (1.22)	44,894 (8.69)
		0.2	268 (18.83)	26 (5.90)	43,843 (41.70)	303 (17.00)	12 (3.47)	44,915 (6.72)	217 (16.97)	3 (1.65)	44,927 (5.69)
		0.3	355 (17.44)	55 (9.28)	44,614 (21.37)	324 (17.24)	15 (4.04)	44,923 (5.99)	217 (16.97)	3 (1.65)	44,927 (5.69)
5.5	0.05	0.1	115 (14.07)	21 (5.84)	27,143 (180.48)	135 (15.44)	11 (3.58)	36,960 (136.69)	145 (15.76)	7 (3.03)	40,583 (93.19)
		0.2	204 (17.79)	53 (10.14)	35,036 (144.69)	242 (17.50)	32 (6.82)	41,926 (75.86)	214 (18.19)	16 (4.64)	42,799 (58.30)
		0.3	277 (19.15)	93 (13.34)	39,209 (107.77)	277 (17.56)	44 (8.11)	42,773 (61.15)	214 (18.16)	16 (4.66)	42,803 (58.29)
	0.001	0.1	115 (14.07)	21 (5.84)	27,143 (180.48)	135 (15.44)	11 (3.58)	36,960 (136.69)	145 (15.75)	7 (3.03)	40,584 (93.18)
		0.2	204 (17.79)	53 (10.14)	35,036 (144.69)	245 (17.55)	33 (6.90)	42,001 (74.29)	266 (18.67)	26 (5.99)	43,769 (42.50)
		0.3	277 (19.15)	93 (13.34)	39,209 (107.77)	328 (17.05)	66 (10.37)	43,751 (42.50)	354 (17.08)	55 (9.14)	44,587 (22.19)
7	0.05	0.1	104 (13.43)	27 (7.03)	21,866 (180.53)	122 (14.51)	16 (4.62)	31,240 (173.73)	132 (15.24)	12 (4.02)	35,683 (135.25)
		0.2	185 (17.37)	63 (11.15)	30,218 (171.50)	219 (17.32)	44 (8.28)	38,281 (123.19)	238 (19.02)	36 (7.51)	41,251 (83.33)
		0.3	253 (18.95)	105 (14.65)	35,350 (143.17)	296 (17.54)	81 (12.12)	41,492 (83.11)	293 (18.47)	57 (9.61)	42,771 (58.85)
	0.001	0.1	104 (13.43)	27 (7.03)	21,866 (180.53)	122 (14.51)	16 (4.62)	31,240 (173.73)	132 (15.24)	12 (4.02)	35,683 (135.25)
		0.2	185 (17.37)	63 (11.15)	30,218 (171.50)	219 (17.32)	44 (8.28)	38,281 (123.19)	238 (19.05)	36 (7.52)	41,256 (83.39)
		0.3	253 (18.95)	105 (14.65)	35,350 (143.17)	296 (17.55)	81 (12.13)	41,511 (82.53)	320 (18.09)	70 (10.81)	43,346 (49.87)

**Table 3:** **Average number of voxels for the practically insignificant layer of the ABT method**.

**σ_*noise*_**	**α**	**β**	**50 scans**			**100 scans**			**150 scans**		
			**ES = 1**	**ES = 2**	**ES = 0**	**ES = 1**	**ES = 2**	**ES = 0**	**ES = 1**	**ES = 2**	**ES = 0**
3	0.05	0.1	2 (1.37)	0 (0.38)	65 (8.62)	98 (10.50)	2 (1.49)	1,798 (51.13)	144 (13.65)	2 (1.22)	2,094 (56.41)
		0.2	60 (7.73)	11 (3.37)	1,164 (36.74)	253 (13.69)	13 (3.70)	2,148 (59.82)	317 (15.17)	10 (3.22)	2,152 (58.23)
		0.3	145 (11.10)	40 (7.18)	1,904 (50.30)	350 (12.54)	34 (6.15)	2,173 (60.70)	415 (11.71)	28 (5.29)	2,154 (58.30)
	0.001	0.1	0 (0)	0 (0)	0 (0)	0 (0.08)	0 (0)	0 (0.19)	2 (1.50)	0 (0.19)	3 (1.64)
		0.2	0 (0.06)	0 (0.04)	0 (0.17)	17 (3.84)	2 (1.25)	14 (3.80)	127 (10.38)	7 (2.77)	28 (5.44)
		0.3	0 (0.44)	0 (0.34)	0 (1.03)	92 (9.90)	19 (4.53)	31 (5.57)	226 (12.28)	25 (5.11)	30 (5.66)
5.5	0.05	0.1	0 (0)	0 (0)	0 (0)	0 (0)	0 (0)	0 (0.06)	0 (0.23)	0 (0.08)	1 (1.11)
		0.2	0 (0)	0 (0)	0 (0)	3 (1.66)	1 (0.89)	75 (8.50)	52 (7.16)	10 (3.17)	970 (33.99)
		0.3	0 (0.25)	0 (0.20)	0 (1.58)	51 (6.61)	22 (5.02)	978 (34.62)	140 (10.85)	39 (6.96)	1,783 (49.35)
	0.001	0.1	0 (0)	0 (0)	0 (0)	0 (0)	0 (0)	0 (0)	0 (0)	0 (0)	0 (0)
		0.2	0 (0)	0 (0)	0 (0)	0 (0)	0 (0)	0 (0)	0 (0)	0 (0)	0 (0)
		0.3	0 (0)	0 (0)	0 (0)	0 (0)	0 (0)	0 (0)	0 (0)	0 (0)	0 (0)
7	0.05	0.1	0 (0)	0 (0)	0 (0)	0 (0)	0 (0)	0 (0)	0 (0)	0 (0)	0 (0)
		0.2	0 (0)	0 (0)	0 (0)	0 (0)	0 (0)	0 (0.10)	0 (0.39)	0 (0.24)	5 (2.06)
		0.3	0 (0)	0 (0)	0 (0.04)	1 (0.74)	0 (0.55)	19 (4.39)	27 (5.44)	13 (3.66)	576 (25.02)
	0.001	0.1	0 (0)	0 (0)	0 (0)	0 (0)	0 (0)	0 (0)	0 (0)	0 (0)	0 (0)
		0.2	0 (0)	0 (0)	0 (0)	0 (0)	0 (0)	0 (0)	0 (0)	0 (0)	0 (0)
		0.3	0 (0)	0 (0)	0 (0)	0 (0)	0 (0)	0 (0)	0 (0)	0 (0)	0 (0)

**Table 4:** **Average number of voxels for the uncertainty layer of the ABT method**.

**σ_*noise*_**	**α**	**β**	**50 scans**			**100 scans**			**150 scans**		
			**ES = 1**	**ES = 2**	**ES = 0**	**ES = 1**	**ES = 2**	**ES = 0**	**ES = 1**	**ES = 2**	**ES = 0**
3	0.05	0.1	64 (8.22)	8 (3.10)	2,020 (52.66)	0 (0)	0 (0)	0 (0)	0 (0)	0 (0)	0 (0)
		0.2	2 (1.53)	1 (0.70)	32 (5.72)	0 (0)	0 (0)	0 (0)	0 (0)	0 (0)	0 (0)
		0.3	0 (0.09)	0 (0.04)	0 (0.13)	0 (0)	0 (0)	0 (0)	0 (0)	0 (0)	0 (0)
	0.001	0.1	311 (13.15)	150 (14.66)	4,156 (88.17)	160 (11.31)	12 (3.62)	345 (20.45)	48 (7.04)	1 (1.14)	33 (5.96)
		0.2	190 (14.11)	131 (13.11)	1,069 (40.70)	21 (4.43)	3 (1.89)	8 (2.88)	0 (0.06)	0 (0)	0 (0)
		0.3	103 (11.75)	102 (11.16)	297 (19.78)	0 (0.41)	0 (0.26)	0 (0.13)	0 (0)	0 (0)	0 (0)
5.5	0.05	0.1	261 (12.45)	155 (15.18)	15,568 (152.94)	142 (10.77)	34 (6.71)	5,821 (98.96)	69 (8.34)	9 (3.10)	2,220 (55.99)
		0.2	171 (11.37)	123 (12.71)	7675 (112.27)	36 (5.79)	12 (3.33)	855 (31.81)	0 (0.49)	0 (0.22)	4 (1.90)
		0.3	99 (9.45)	83 (9.90)	3,502 (71.21)	1 (0.76)	0 (0.47)	8 (2.78)	0 (0)	0 (0)	0 (0)
	0.001	0.1	389 (13.21)	419 (11.21)	17769 (179.47)	351 (13.85)	263 (17.80)	7,963 (135.89)	320 (12.89)	155 (14.89)	4,343 (92.29)
		0.2	300 (16.61)	387 (11.97)	9,876 (143.45)	242 (14.98)	241 (15.86)	2,922 (73.26)	200 (13.75)	136 (13.64)	1,158 (41.55)
		0.3	227 (17.71)	347 (12.37)	5,700 (106.53)	159 (13.94)	208 (14.23)	1,173 (41.07)	112 (11.22)	107 (11.10)	340 (20.91)
7	0.05	0.1	307 (12.29)	234 (16.29)	20,846 (156.61)	221 (12.51)	98 (12.27)	11,541 (138.06)	162 (11.31)	47 (8.08)	7,120 (102.51)
		0.2	226 (12.71)	198 (14.10)	12,493 (141.28)	124 (10.18)	70 (9.50)	4,500 (84.68)	57 (7.58)	22 (5.31)	1,552 (44.64)
		0.3	157 (11.82)	156 (12.20)	7,361 (109.52)	47 (6.70)	33 (5.82)	1,289 (40.32)	2 (1.32)	1 (1.02)	32 (5.57)
	0.001	0.1	405 (12.89)	452 (8.63)	23,046 (179.77)	379 (13.48)	377 (14.47)	13,683 (172.93)	360 (13.52)	296 (16.95)	9,244 (134.50)
		0.2	324 (16.66)	416 (10.60)	14,693 (170.28)	282 (15.81)	348 (13.17)	6,642 (122.34)	254 (16.52)	271 (15.51)	3,671 (82.60)
		0.3	255 (18.09)	374 (12.83)	9,561 (141.90)	205 (15.78)	311 (12.50)	3,412 (81.55)	172 (15.10)	237 (13.75)	1,580 (49.01)

**Table 5:** **Effect sizes (η^**2**^ × 100%) for the main and interaction effects of the four factors that constitute the simulation design explaining the variability in the scientifically irrelevant, scientifically relevant and noise voxels, respectively, for each layer in the LSPM in the simulation study**.

**Parameter**	**Active**			**Inactive**			**Uncertain**			**Practically insignificant**		
	**ES = 1**	**ES = 2**	**ES = 0**	**ES = 1**	**ES = 2**	**ES = 0**	**ES = 1**	**ES = 2**	**ES = 0**	**ES = 1**	**ES = 2**	**ES = 0**
**MAIN EFFECTS**												
Scans	6.02	19.03	3.33	0.53	13.99	26.44	13.51	16.20	25.86	8.59	3.17	4.30
σ_*noise*_	12.76	37.78	14.31	5.88	30.31	32.09	30.58	32.66	31.87	33.31	15.12	24.35
α	46.38	29.20	54.68	11.59	1.09	0.41	27.04	33.83	1.49	9.45	8.86	25.32
β	7.65	0.11	1.82	40.72	31.42	20.16	18.38	1.44	20.02	8.03	23.35	3.10
**INTERACTION EFFECTS**												
Scans × σ_*noise*_	0.27	2.27	1.12	10.38	0.64	8.87	0.13	2.24	8.81	9.70	1.18	1.91
Scans × α	0.02	1.07	2.99	2.21	0.03	0.07	0.01	1.24	0.08	1.80	0.22	4.15
Scans × β	2.15	0.02	0.12	0.59	3.28	3.10	0.27	0.15	3.08	2.26	3.14	0.20
σ_*noise*_ × α	3.28	8.25	13.88	13.09	0.33	0.38	1.91	9.55	0.38	10.63	2.69	23.62
σ_*noise*_ × β	8.36	0.06	0.52	3.33	9.08	7.72	1.50	0.42	7.67	8.77	12.15	0.88
α × β	1.72	0.04	1.75	2.23	0.98	0.05	1.01	0.05	0.05	1.81	7.98	2.97

*Effect sizes are based on type II sum of squares. Hence, effect sizes of main effects do not account for interactions*.

#### 3.1.1. Localizer task parameters

An increasing amount of noise, and as a consequence a larger SE, led to a decrease of signal voxels (ES = 1, η^2^ = 12.76%; ES = 2, η^2^ = 37.78%) in the active layer and an increase in noise voxels (ES = 0, η^2^ = 14.31%). For the scientifically irrelevant voxels, this decrease was larger with smaller values for β (η^2^ = 8.36%), while for the scientifically relevant voxels this decrease was larger for smaller values of α (η^2^ = 8.25%). However, the decrease in scientifically irrelevant voxels (ES = 1) was larger than that of the scientifically relevant voxels (ES = 2). The increase in noise voxels was larger with larger values for α (η^2^ = 13.88%). In the inactive layer, the number of scientifically irrelevant (η^2^ = 5.88%) and noise voxels (η^2^ = 32.09%) decreased as the amount of noise increased, while the number of scientifically relevant voxels increased (η^2^ = 30.31%). The decrease in scientifically irrelevant voxels was largest when α = 0.05 and the amount of noise was low (η^2^ = 13.09%). However, this decrease was mainly present when the amount of noise is low and the number of scans is large. For larger amounts of noise and a larger number of scans, the number of scientifically irrelevant voxels increased (η^2^ = 10.38%). For the scientifically relevant voxels, the increase was larger when β increased (η^2^ = 9.08%). The decrease in noise voxels was large when the number of scans was smaller (η^2^ = 8.87%) or when β was smaller (η^2^ = 7.72%). Finally, increasing the amount of noise reduced the number of practically insignificant voxels (all η^2^s > 15.12%) while increasing the number of uncertain voxels (all η^2^s > 30.58%). The latter results were expected since a larger SE puts *t*_β_ to the left, inducing the scenario where the uncertainty layer becomes possible for a certain voxel. Specifically, for the practically insignificant layer, the decrease in the number of scientifically irrelevant voxels was largest when the number of scans was large (η^2^ = 9.70%). The decrease in scientifically irrelevant voxels was also largest when the amount of noise was low and α increased (η^2^ = 10.63%) or β increased (η^2^ = 8.77%). The number of scientifically relevant voxels in the practically insignificant layer decreased more with larger values for β as well (η^2^ = 12.15%). The number of noise voxels in the practically insignificant layer decreased more when α=0.05 (η^2^ = 23.62%). In the uncertainty layer, the decrease in scientifically relevant voxels caused by the increase in noise was larger with more stringent values for α (η^2^ = 9.55%). For the noise voxels, the influence of the noise was larger when there were less scans (η^2^ = 8.81%) and smaller values for β were chosen (η^2^ = 7.67%).

Increasing the number of scans reduced the number of noise voxels in the active layer (η^2^ = 3.33%). More importantly, the number of scientifically relevant voxels increased (η^2^ = 19.03%), while the number of scientifically irrelevant voxels showed an initial increase that changed into a decrease with 150 scans for multiple scenarios (η^2^ = 6.02%). In the inactive layer, the number of signal voxels decreased (ES = 1, η^2^ = 0.53%; ES = 2, η^2^ = 13.99%) while the number of noise voxels increased as the number of scans was larger (η^2^ = 26.44%). The number of scientifically relevant voxels (η^2^ = 3.17%) and noise voxels (η^2^ = 4.30%) increased in the practically insignificant layer. Additionally, the number of scientifically relevant voxels decreased when the amount of noise was low (η^2^ = 9.70%). In the uncertainty layer, the proportion of all type of voxels decreased by adding more scans (η^2^s > 13.51%). Again, these latter findings were expected, as increasing the number of scans decreases the SE for a voxel, inducing the scenario where the practically insignificant layer becomes possible for a certain voxel.

#### 3.1.2. Data analysis parameters

As α increases all types of voxels increase in the active layer (all η^2^s > 29.20%). Furthermore, the average increase in scientifically relevant voxels (η^2^ = 29.20%) is larger than that of scientifically irrelevant voxels (η^2^ = 46.38%). In the inactive layer, the number of noise voxels decreases as α increases (η^2^ = 0.41%), as do the signal voxels (ES = 1, η^2^ = 11.59%; ES = 2, η^2^ = 1.09%). This average decrease is larger for the scientifically relevant voxels. However, as the amount of noise increases, α does not influence the information in the inactive layer to a great extent. For the practically insignificant layer, increasing α resulted in more voxels of all types (all η^2^s > 8.86%), while the opposite was true in the uncertainty layer (ES = 1, η^2^ = 27.04%; ES = 2, η^2^ = 33.83%; ES = 0, η^2^ = 1.49%). This trend was more pronounced for the scientifically irrelevant voxels (η^2^ = 27.04% in the uncertainty layer; η^2^ = 9.45% in the practically insignificant layer) and the scientifically relevant voxels (η^2^ = 33.83% in the uncertainty layer; η^2^ = 8.86% in the practically insignificant layer). This increase of scientifically relevant voxels in the practically insignificant layer was larger for larger β values as well (η^2^ = 7.98%). These results were expected, since larger α values locate *t*_α_ more to the left, increasing the probability of practically insignificant voxels, as mentioned above. In some cases interactions between σ_*noise*_ and α were present with a medium or larger effect size. These interactions are already discussed in the results section on localizer task parameters.

Finally, increasing β led to a decrease in the number of active voxels (ES = 1, η^2^ = 7.65%; ES = 2, η^2^ = 0.11%; ES = 0, η^2^ = 1.82%), especially for both scientifically relevant and noise voxels when the amount of noise was low. When the amount of noise increased, β does not show that much influence on the information in the active layer. This is symmetrical to the influence of α in the inactive layer. An increase in β also increased the number of inactive voxels (all η^2^s > 20.16%). The number of scientifically irrelevant (η^2^ = 40.72%) and noise voxels (η^2^ = 20.16%) increased more than the number of scientifically relevant voxels (η^2^ = 31.42%). In the scenarios where practically insignificant voxels were found, the average number increased as β increased (ES = 1, η^2^ = 8.03%; ES = 2, η^2^ = 23.35%; ES = 0, η^2^ = 3.10%), especially the average number of scientifically irrelevant voxels. For the scientifically relevant voxels, this increase for larger values of β was larger as α increased (η^2^ = 7.98%). Finally, in the uncertainty layer, all types of voxels decreased as β increased (ES = 1, η^2^ = 18.38%; ES = 2, η^2^ = 1.44%; ES = 0, η^2^ = 20.02%), most of all the scientifically irrelevant and the number of noise voxels.

#### 3.1.3. Discussion

These simulations show that ABT is able to provide valuable information for the definition of fROIs. First of all, the results show that the active layer largely consists of scientifically relevant voxels which are truthfully part of the fROI. Secondly, what is present in the inactive layer is either noise or activation that is not relevant in the research context, which makes dismissing the voxels in the inactive layer a valid decision. These findings are especially true with α at 0.001, larger β values, a moderate amount of noise and a high number of scans. Combined, these results show direct control of FPs and FNs through α and β, respectively, since α mainly influences the size of the active layer while β mainly influences the size of the inactive layer. Additionally, it shows that by controlling the localizer task parameters, i.e., a slight increase of the number of scans in the localizer task, using a design with more variance and avoiding noise as much as possible, the researcher can substantially improve the scientific relevance of the information given by the ABT method. This trend can be seen in all rows of Figure 4.

The practically insignificant layer is mainly influenced by localizer task parameters, i.e., low amounts of noise and high number of scans. For all parameter configurations, only a very small portion of the practically insignificant layer consisted of truly active voxels. Both conclusions are clearly visible in the upper row of Figure 4. The opposite parameter configurations, combined with stringent α and small β values showed an increase of uncertain voxels. As shown in the middle and bottom row of Figure 4, in multiple scenarios over half of the 515 scientifically relevant signal voxels were classified as uncertain. However, this comes with the inclusion of noise voxels and scientifically irrelevant voxels and proves to be a trade-off researchers have to consider in the context of their specific localizer task. If the parameters of the localizer task cause the SE to be large, the uncertainty layer proves to be an asset in extracting all relevant information from the task. In the contrary situation, adding the uncertainty layer including its noise voxels can be disadvantageous, since most scientifically relevant voxels are already categorized as active. In conclusion, as seen in Figure 4 ABT does not lead to a large loss of information as compared to NHST, since the number of practically insignificant voxels is small in realistic functional localizer conditions. Additionally, there is a gain of information in the uncertainty layer.

Finally, it is possible that the a priori defined scientifically relevant ES, the mean of the alternative distribution in ABT, misrepresents the true underlying ES of the to be defined fROI. The simulation results can shed light on what happens when the scientifically relevant ES is overestimated in the situation where we specify μ_Δ_1__ to 1.5 % BOLD signal change, but the true underlying ES within the fROI is at least 1% BOLD signal change. The results show that a significant proportion of the now scientifically relevant voxels (ES = 1 and ES = 2) ends up in the active layer, especially when the number of scans increases, the amount of noise is low and α and β are small. As a result of the misspecification, a large part of these voxels can also be found in the practically insignificant layer. Under opposite parameter configurations these voxels are found in the uncertainty layer with stringent values for α, larger values for β, a larger amount of noise and fewer scans. If the researcher follows the guidelines to ensure maximum information in the active layer mentioned above, the results show a loss of relevant information if μ_Δ_1__ is an overestimation of the true scientifically relevant ES, since the voxels with ES = 1 are now situated mainly in the inactive layer. Nonetheless, a substantial part of the now scientifically relevant voxels can be found in the uncertainty layer under these parameter configurations. While overestimation leads to a loss of information, logically an underestimation of the true ES could result in the detection of more regions. This was not explored in the current simulation study.

### 3.2. Study 2: real data example

**Figure 5:**
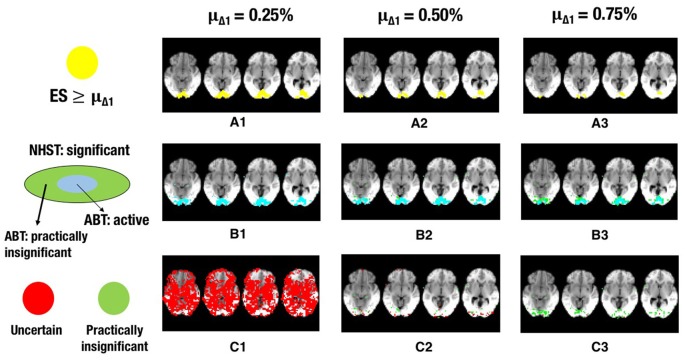
**The illustration shows a visual representation of the reference map of effect sizes and the different layers for one step in the cross-validation procedure. (A)** Visual representation of the ground truth of effect sizes, with the red area indicating effect sizes ≥ 0.25% **(A1)**, effect sizes ≥ 0.50% **(A2)** or effect sizes ≥ 0.75% **(A3)** BOLD signal change. **(B)** Visual representation of the active layer (light blue) and the significant (green) layer for μ_Δ_1__ = 0.25% **(B1)**, μ_Δ_1__ = 0.50% **(B2)**, or μ_Δ_1__ = 0.75% **(B3)** BOLD signal change. **(C)** Visual representation of the uncertainty (red) and the practically insignificant (green) layer, for μ_Δ_1__ = 0.25% **(C1)**, μ_Δ_1__ = 0.50% **(C2)**, or μ_Δ_1__ = 0.75% **(C3)** BOLD signal change. α = 0.001 and β = 0.1 in all scenarios shown.

**Figure 6:**
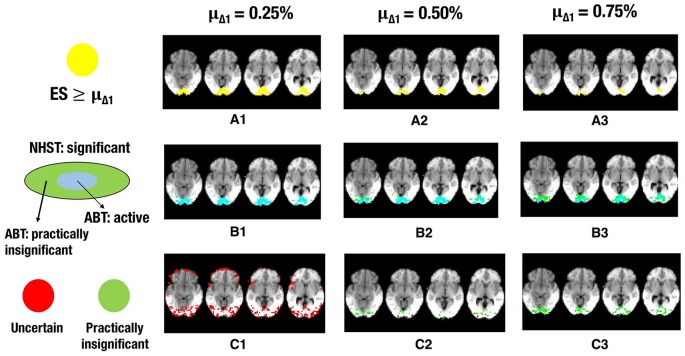
**The illustration shows a visual representation of the reference map of effect sizes and the different layers for one step in the cross-validation procedure. (A)** Visual representation of the ground truth of effect sizes, with the red area indicating effect sizes ≥ 0.25% **(A1)**, effect sizes ≥ 0.50% **(A2)** or effect sizes ≥ 0.75% **(A3)** BOLD signal change. **(B)** Visual representation of the active layer (light blue) and the significant (green) layer for μ_Δ_1__ = 0.25% **(B1)**, μ_Δ_1__ = 0.50% **(B2)**, or μ_Δ_1__ = 0.75% **(B3)** BOLD signal change. **(C)** Visual representation of the uncertainty (red) and the practically insignificant (green) layer, for μ_Δ_1__ = 0.25% **(C1)**, μ_Δ_1__ = 0.50% **(C2)**, or μ_Δ_1__ = 0.75% (C3) BOLD signal change. α = 0.001 and β = 0.2 in all scenarios shown.

**Figure 7:**
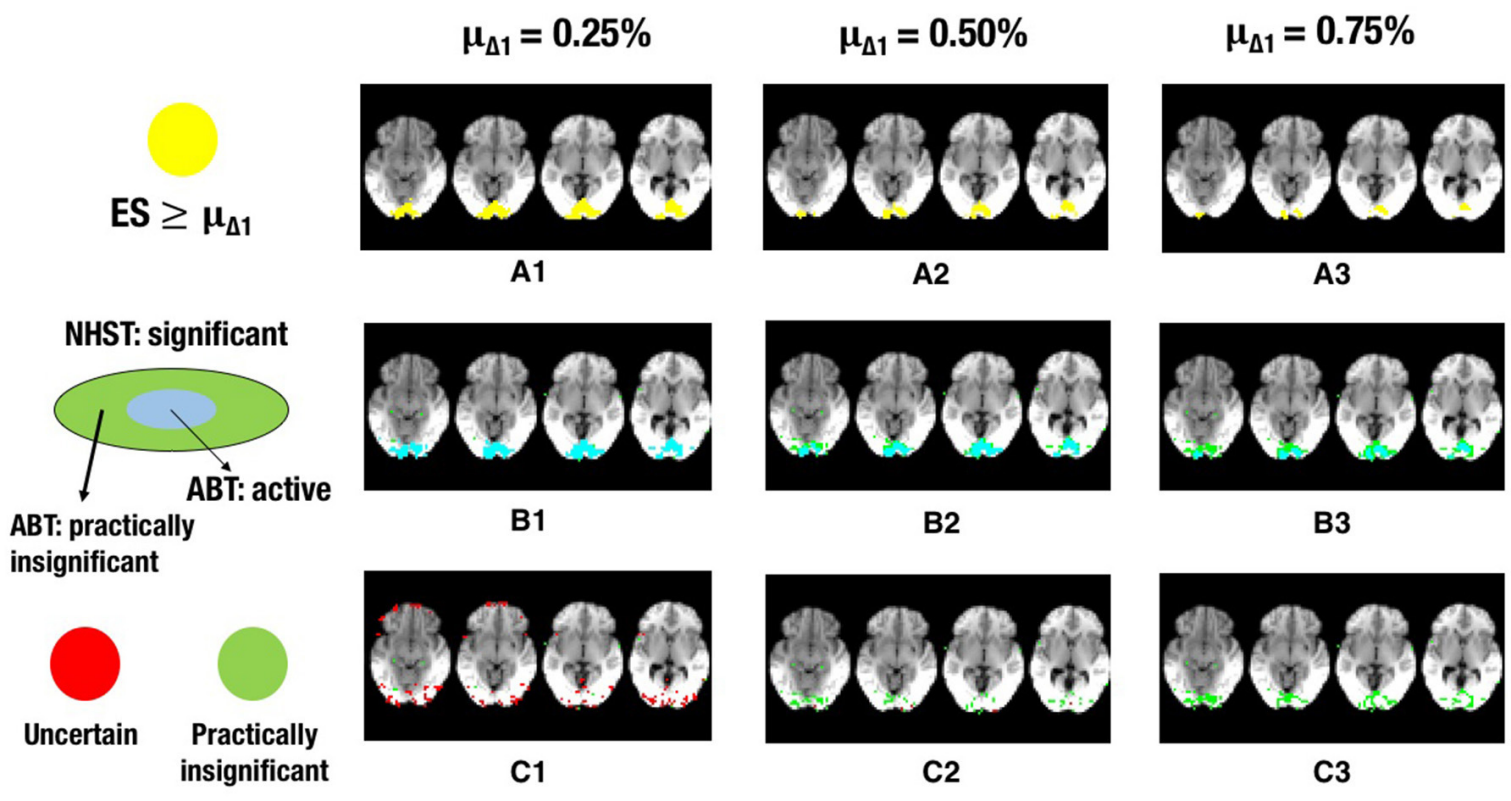
**The illustration shows a visual representation of the reference map of effect sizes and the different layers for one step in the cross-validation procedure. (A)** Visual representation of the ground truth of effect sizes, with the red area indicating effect sizes ≥ 0.25% **(A1)**, effect sizes ≥ 0.50% **(A2)**, or effect sizes ≥ 0.75% **(A3)** BOLD signal change. **(B)** Visual representation of the active layer (light blue) and the significant (green) layer for μ_Δ_1__ = 0.25% **(B1)**, μ_Δ_1__ = 0.50% **(B2)**, or μ_Δ_1__ = 0.75% **(B3)** BOLD signal change. **(C)** Visual representation of the uncertainty (red) and the practically insignificant (green) layer, for μ_Δ_1__ = 0.25% **(C1)**, μ_Δ_1__ = 0.50% **(C2)**, or μ_Δ_1__ = 0.75% **(C3)** BOLD signal change. α = 0.001 and β = 0.3 in all scenarios shown.

The results for ABT can be found in Tables 6, 7. The results for uncorrected and FDR-corrected NHST can be found in Tables 8, 9. The trends in the results for ABT with the α threshold for the null distribution and for ABT with the threshold using FDR were very similar. As a consequence, the following results section will focus on the results found with uncorrected NHST, as this method was also used in the simulation study. Additionally, information on the distribution of the ground truth ESs in each layer, in terms of means, medians and standard deviations were also computed and can be found in Tables A1, A2 in the Appendix (Supplementary Material). As an illustration, a visual representation of the ground truth of ESs and the different layers for one step of the cross-validation procedure with α = 0.001 and β = 0.1, 0.2 and 0.3 is shown in Figures 5–**7**, respectively.

**Table 6:** **Results in the visual + letter/number discrimination (Gonzalez-Castillo et al., 2012) task analyzed with the ABT method**.

**μ_Δ_1__**	**α**	**β**	**Active**		**Inactive**		**Uncertainty**		**Practically insignificant**	
			**RDR**	**LDR**	**RDR**	**LDR**	**RDR**	**LDR**	**RDR**	**LDR**
0.25	0.05	0.1	0.95 (0.036)	0.16 (0.070)	0.26 (0.097)	0.9803 (0.008)	0.04 (0.031)	0.001 (0.001)	0 (0)	0 (0)
		0.2	0.95 (0.036)	0.27 (0.094)	0.86 (0.068)	0.9838 (0.002)	0.03 (0.021)	0.03 (0.027)	5.57e^−3^ (0.001)	3.44e^−3^ (0.001)
		0.3	0.94 (0.048)	0.46 (0.012)	0.88 (0.063)	0.9837 (0.002)	0.01 (0.010)	0.08 (0.077)	0.01 (0.017)	0.004 (0.007)
	0.001	0.1	0.87 (0.081)	0.43 (0.139)	0.25 (0.097)	0.9803 (0.008)	0.13 (0.077)	0.003 (0.002)	0 (0)	0 (0)
		0.2	0.87 (0.081)	0.47 (0.141)	0.92 (0.041)	0.9834 (0.002)	0.11 (0.068)	0.04 (0.037)	0 (0)	0 (0)
		0.3	0.87 (0.083)	0.58 (0.131)	0.96 (0.024)	0.9834 (0.002)	0.09 (0.053)	0.13 (0.097)	0.003 (0.007)	0.009 (0.023)
0.50	0.05	0.1	0.98 (0.019)	0.32 (0.082)	0.88 (0.062)	0.9843 (0.002)	0.002 (0.004)	0.01 (0.035)	0.006 (0.011)	7.65e^−3^ (0.002)
		0.2	0.96 (0.044)	0.45 (0.106)	0.88 (0.061)	0.9843 (0.002)	7.09^−3^ (0.001)	0.04 (0.159)	0.03 (0.036)	0.003 (0.005)
		0.3	0.93 (0.070)	0.54 (0.121)	0.88 (0.061)	0.9843 (0.002)	5.46^−5^ (0.0001)	0.008 (0.063)	0.06 (0.062)	0.006 (0.008)
	0.001	0.1	0.96 (0.035)	0.36 (0.090)	0.96 (0.021)	0.9843 (0.002)	0.03 (0.044)	0.05 (0.044)	0.003 (0.007)	0.002 (0.005)
		0.2	0.94 (0.050)	0.47 (0.109)	0.96 (0.020)	0.9842 (0.002)	0.02 (0.019)	0.11 (0.101)	0.02 (0.028)	0.01 (0.016)
		0.3	0.92 (0.071)	0.56 (0.120)	0.97 (0.020)	0.9842 (0.002)	0.01 (0.014)	0.15 (0.150)	0.04 (0.051)	0.02 (0.026)
0.75	0.05	0.1	0.97 (0.042)	0.35 (0.098)	0.87 (0.061)	0.9844 (0.002)	0 (0)	0 (0)	0.03 (0.040)	0.001 (0.002)
		0.2	0.94 (0.068)	0.44 (0.120)	0.87 (0.061)	0.9844 (0.002)	0 (0)	0 (0)	0.06 (0.066)	0.003 (0.004)
		0.3	0.91(0.098)	0.51 (0.129)	0.87 (0.061)	0.9843 (0.002)	0 (0)	0 (0)	0.09 (0.096)	0.004 (0.008)
	0.001	0.1	0.96 (0.044)	0.35 (0.098)	0.96 (0.020)	0.9844 (0.002)	0.007 (0.014)	0.06 (0.102)	0.02 (0.037)	0.004 (0.008)
		0.2	0.94 (0.068)	0.45 (0.119)	0.96 (0.020)	0.9844 (0.002)	0.004 (0.009)	0.11 (0.193)	0.05 (0.063)	0.008 (0.012)
		0.3	0.91 (0.098)	0.52 (0.128)	0.96 (0.020)	0.9843 (0.002)	0.003 (0.006)	0.17 (0.32)	0.08 (0.092)	0.01 (0.017)

*For the active, uncertainty and practically insignificant layer the mean proportion of scientifically relevant voxels in the ground truth that were categorized in the respective layer was computed over simulations, i.e., the reference detection rate (RDR; ≥ μ_Δ_1__/reference). Additionally, the mean proportion of voxels in the respective layer that were truthfully scientifically relevant was also calculated over the 100 cross-validation steps, i.e., the layer detection rate (LDR; ≥ μ_Δ_1__/layer). For the inactive layer, the mean proportion of scientifically irrelevant voxels in the ground truth that was categorized in the layer was computed over simulations, i.e., the RDR for the inactive layer (< μ_Δ_1__/reference). Additionally, the mean proportion of voxels in the inactive layer that were truthfully scientifically irrelevant was also calculated over the 100 cross-validation steps, i.e., the LDR for the inactive layer (< μ_Δ_1__/layer). Standard deviations over all cross-validation steps are between brackets*.

**Table 7:** **Results in the visual + letter/number discrimination (Gonzalez-Castillo et al., 2012) task analyzed with the ABT method where the threshold under the null distribution was defined by FDR control (controlled at level ***q***)**.

**μ_Δ_1__**	***q***	**β**	**Active**		**Inactive**		**Uncertainty**		**Practically insignificant**	
			**RDR**	**LDR**	**RDR**	**LDR**	**RDR**	**LDR**	**RDR**	**LDR**
0.25	0.05	0.1	0.81 (0.111)	0.56 (0.150)	0.26 (0.097)	0.9803 (0.008)	0.19 (0.108)	0.005 (0.003)	0 (0)	0 (0)
		0.2	0.81 (0.111)	0.58 (0.152)	0.92 (0.040)	0.9833 (0.002)	0.17 (0.099)	0.08 (0.045)	0 (0)	0 (0)
		0.3	0.81 (0.112)	0.65 (0.137)	0.97 (0.019)	0.9833 (0.002)	0.15 (0.083)	0.15 (0.109)	0.001 (0.003)	0.01 (0.030)
	0.01	0.1	0.76 (0.126)	0.64 (0.147)	0.26 (0.097)	0.9803 (0.008)	0.23 (0.123)	0.006 (0.003)	0 (0)	0 (0)
		0.2	0.76 (0.126)	0.66 (0.146)	0.92 (0.040)	0.9832 (0.002)	0.22 (0.114)	0.07 (0.050)	0 (0)	0 (0)
		0.3	0.76 (0.126)	0.70 (0.134)	0.97 (0.017)	0.9832 (0.002)	0.19 (0.099)	0.18 (0.115)	0.0007 (0.002)	0.001 (0.030)
0.50	0.05	0.1	0.93 (0.052)	0.40 (0.100)	0.97 (0.015)	0.9842 (0.002)	0.05 (0.043)	0.06 (0.051)	0.002 (0.005)	0.003 (0.008)
		0.2	0.92 (0.060)	0.49 (0.115)	0.98 (0.014)	0.9841 (0.002)	0.04 (0.035)	0.13 (0.096)	0.01 (0.022)	0.01 (0.024)
		0.3	0.90 (0.077)	0.57 (0.124)	0.98 (0.013)	0.984 (0.002)	0.03 (0.030)	0.20 (0.140)	0.03 (0.044)	0.02 (0.039)
	0.01	0.1	0.91 (0.065)	0.42 (0.104)	0.98 (0.012)	0.9841 (0.002)	0.08 (0.057)	0.08 (0.059)	0.001 (0.003)	0.004 (0.010)
		0.2	0.90 (0.071)	0.51 (0.116)	0.98 (0.010)	0.984 (0.002)	0.06 (0.047)	0.15 (0.107)	0.01 (0.018)	0.01 (0.027)
		0.3	0.88 (0.085)	0.58 (0.124)	0.98 (0.010)	0.984 (0.002)	0.05 (0.040)	0.23 (0.152)	0.03 (0.039)	0.03 (0.045)
0.75	0.05	0.1	0.95 (0.049)	0.36 (0.102)	0.97 (0.013)	0.9843 (0.002)	0.02 (0.025)	0.08 (0.104)	0.02 (0.036)	0.01 (0.013)
		0.2	0.93 (0.070)	0.45 (0.121)	0.97 (0.013)	0.9843 (0.002)	0.01 (0.019)	0.15 (0.21)	0.04 (0.061)	0.02 (0.018)
		0.3	0.90 (0.099)	0.52 (0.129)	0.97 (0.013)	0.9842 (0.002)	0.01 (0.016)	0.21 (0.296)	0.07 (0.090)	0.03 (0.027)
	0.01	0.1	0.94 (0.055)	0.37 (0.104)	0.98 (0.010)	0.9842 (0.002)	0.03 (0.032)	0.10 (0.157)	0.02 (0.034)	0.01 (0.016)
		0.2	0.92 (0.073)	0.46 (0.123)	0.98 (0.010)	0.9842 (0.002)	0.02 (0.025)	0.17 (0.157)	0.04 (0.058)	0.02 (0.022)
		0.3	0.89 (0.101)	0.52 (0.131)	0.98 (0.010)	0.9842 (0.002)	0.02 (0.022)	0.23 (0.226)	0.07 (0.086)	0.03 (0.031)

*For the active, uncertainty and practically insignificant layer the mean proportion of scientifically relevant voxels in the ground truth that were categorized in the respective layer was computed over simulations, i.e., the reference detection rate (RDR; ≥ μ_Δ_1__/reference). Additionally, the mean proportion of voxels in the respective layer that were truthfully scientifically relevant was also calculated over the 100 cross-validation steps, i.e., the layer detection rate (LDR; ≥ μ_Δ_1__/layer). For the inactive layer, the mean proportion of scientifically irrelevant voxels in the ground truth that was categorized in the layer was computed over simulations, i.e., the RDR for the inactive layer (< μ_Δ_1__/reference). Additionally, the mean proportion of voxels in the inactive layer that were truthfully scientifically irrelevant was also calculated over the 100 cross-validation steps, i.e., the LDR for the inactive layer (< μ_Δ_1__/layer). Standard deviations over all cross-validation steps are between brackets*.

**Table 8:** **Results in the visual + letter/number discrimination (Gonzalez-Castillo et al., 2012) task analyzed with NHST**.

**μ_Δ_1__**	**α**	**Significant**		**Non-significant**	
		**RDR**	**LDR**	**RDR**	**LDR**
0.25	0.05	0.95 (0.036)	0.16 (0.070)	0.86 (0.061)	0.9833 (0.002)
	0.001	0.87 (0.081)	0.43 (0.139)	0.97 (0.019)	0.9819 (0.002)
0.50	0.05	0.99 (0.012)	0.07 (0.033)	0.88 (0.061)	0.9842 (0.002)
	0.001	0.99 (0.033)	0.21 (0.079)	0.97 (0.019)	0.9840 (0.002)
0.75	0.05	0.998 (0.005)	0.03 (0.016)	0.87 (0.061)	0.9843 (0.002)
	0.001	0.99 (0.021)	0.10 (0.042)	0.96 (0.020)	0.9843 (0.002)

*For the significant layer the mean proportion of scientifically relevant voxels in the ground truth that were categorized as significant was computed over simulations, i.e., the reference detection rate (RDR; ≥ μ_Δ_1__/reference). Additionally, the mean proportion of voxels in the significant layer that were truthfully scientifically relevant was also calculated over simulations, i.e., the layer detection rate (LDR; ≥ μ_Δ_1__/layer). For the non-significant layer, the mean proportion of scientifically irrelevant voxels in the ground truth that was categorized in the layer was computed over simulations, i.e., the RDR for the non-significant layer (< μ_Δ_1__/reference). Additionally, the mean proportion of voxels in the non-significant layer that were truthfully scientifically irrelevant was also calculated over simulations, i.e., the LDR for the non-significant layer (< μ_Δ_1__/layer). Standard deviations over all cross-validation steps are between brackets*.

**Table 9:** **Results in the visual + letter/number discrimination (Gonzalez-Castillo et al., 2012) task analyzed with NHST with FDR control (controlled at level ***q***)**.

**μ_Δ_1__**	***q***	**Significant**		**Non-significant**	
		**RDR**	**LDR**	**RDR**	**LDR**
0.25	0.05	0.81 (0.111)	0.56 (0.150)	0.985 (0.012)	0.981 (0.002)
	0.01	0.76 (0.126)	0.64 (0.147)	0.99 (0.009)	0.98 (0.003)
0.50	0.05	0.93 (0.051)	0.28 (0.100)	0.978 (0.013)	0.984 (0.002)
	0.01	0.91 (0.065)	0.34 (0.108)	0.983 (0.010)	(0.002)
0.75	0.05	0.97 (0.033)	0.14 (0.056)	0.975 (0.013)	0.984 (0.002)
	0.01	0.96 (0.041)	0.17 (0.064)	0.98 (0.010)	0.984 (0.002)

*For the significant layer the mean proportion of scientifically relevant voxels in the ground truth that were categorized as significant was computed over simulations, i.e., the reference detection rate (RDR; ≥ μ_Δ_1__/reference). Additionally, the mean proportion of voxels in the significant layer that were truthfully scientifically relevant was also calculated over simulations, i.e., the layer detection rate (LDR; ≥ μ_Δ_1__/layer). For the non-significant layer, the mean proportion of scientifically irrelevant voxels in the ground truth that was categorized in the layer was computed over simulations, i.e., the RDR for the non-significant layer (< μ_Δ_1__/reference). Additionally, the mean proportion of voxels in the non-significant layer that were truthfully scientifically irrelevant was also calculated over simulations, i.e., the LDR for the non-significant layer (< μ_Δ_1__/layer). Standard deviations over all cross-validation steps are between brackets*.

Increasing α resulted in a larger RDR in the active layer of the LSPM and the significant layer for NHST, while the RDR decreased in the inactive layer and the non-significant layer of NHST, as was found in the simulations. However, this increase in the active and significant layer was paired with a decrease in the LDR. The RDR in the uncertainty layer decreased as α increased. Similar to the active and significant layers, this was paired with a decrease of the LDR. The opposite pattern was found for the practically insignificant layer, but only when μ_Δ_1__ ≥ 0.50% BOLD signal change, which locates the cut-off under *H*_1_, *t*_β_ more to the right. Again, this corroborates the findings of the simulation study.

Similar trends for β as found in the simulation study were also found in the real data example. An increase in β led to a decrease of the RDR in the active layer and an increase in the inactive layer. The LDR increased in the active layer. The RDR in the uncertainty layer decreased as β increased, while it increased in the practically insignificant layer. An increase in β also led to an increase of the LDR in the uncertainty layer and the practically insignificant layer (again, only when μ_Δ_1__ ≥ 0.50% BOLD signal change).

As μ_Δ_1__ increases, i.e., only larger ESs are a priori deemed scientifically relevant, a larger RDR was found in the significant and active layer. This increase was associated with an initial increase followed by a decrease of the LDR of the active layer. The same initial increase and subsequent decrease was found for the RDR in the inactive or non-significant layer. This proportion was substantially large in close to all scenarios, except when the smallest μ_Δ_1__ and small β values were used. The RDR in the uncertainty layer decreased as μ_Δ_1__ increased, since *t*_β_ is located more to the right again, while it increased in the practically insignificant layer. The LDR in the uncertainty layer increased when μ_Δ_1__ increased, but only when α = 0.001. In the practically insignificant layer, this first increased and then decreased somewhat in multiple scenarios.

#### 3.2.1. Discussion

The results of the real data example corroborate conclusions from the simulation study, showing that the ABT method provides valuable information for the definition of fROIs. The active layer of the ABT method captures a proportion of the scientifically relevant ground truth that is either as large or only somewhat smaller as compared to the significant layer of NHST. Additionally, in all scenarios the active layer itself consists of an equal or a larger proportion of voxels that are truthfully scientifically relevant (≥ μ_Δ_1__/layer) as compared to the significant layer of NHST, which shows that the active layer is more specific. This proportion is especially larger with stringent α and β values, as in the simulations, and when the mean of the functionally relevant alternative μ_Δ_1__ is larger than 0.5% BOLD signal change.

Second, as with the simulations, the results here show that what is in the inactive layer can be confidently excluded as part of the fROI, since this layer now largely consists of scientifically irrelevant and noise voxels. The RDR in the inactive layer is never lower than in the non-significant layer. Additionally, the LDR of the inactive layer is almost always equal or higher to that of the non-significant layer, except when β = 0.1 and μ_Δ_1__ is small, again showing that this layer itself consists of more scientifically irrelevant and noise voxels as compared to the non-significant layer. We want to note that this proportion does not vary that much across scenarios since the inactive and non-significant layer are quite large, so small changes in this proportion are not easily detectable.

Next to the active and inactive layer, the ABT also provides information beyond that found with traditional data analysis methods in the uncertainty and practically insignificant layer. As in the simulations, the uncertainty layer can contain large parts of the scientifically relevant ground truth which would be undetected with NHST. This is especially the case when α is stringent and when lower values for β are used, as can be seen in Figure 5. In the scenarios where the RDR in the active layer is the lowest (stringent α and larger β values), a large part of the missing proportion can be found in the uncertainty layer. Again, this comes with the inclusion of noise voxels (see Figure 5), similar to the results in the simulations. As β increases, so does the LDR of the uncertainty layer (see Figures 6, 7). The uncertainty layer itself also consists of a larger number of scientifically relevant voxels than the practically insignificant layer in close to all scenarios, especially when α is stringent and β is larger. As mentioned above, the active layer combined with the practically insignificant equals the significant layer of NHST. However, as in the simulation study, these results show that discarding the practically insignificant layer and including the uncertainty layer instead may increase the amount of scientifically relevant information and may be useful in particular circumstances in which it is detrimental to miss important information, as in the case of defining fROIs.

Finally, the results and Figures 5–7 also show that the ABT adjusts well to the ES that is defined as scientifically relevant. As an a priori larger scientifically relevant ES of interest is defined, the difference between NHST and ABT increases, which is clearly visible in the middle row of Figures 5–7. In this scenario, a substantial part of the regions detected by NHST is not scientifically relevant, while the active layer adjusts to the a priori defined alternative. As μ_Δ_1__ gets larger, the significant layer of NHST is increasingly more divided into an active and practically insignificant layer, the latter containing the scientifically irrelevant regions detected by NHST (see Figures 5–7, middle and bottom row). When a researcher specifies an alternative with a scientifically relevant ES that is rather small, it is shown that a rather large part of the scientifically relevant voxels can be found in the uncertainty layer (see Figures 5, 6, 7C1). With larger values for μ_Δ_1__, the practically insignificant layer can contain valuable information for the definition of the fROI as well. These conclusions can also be drawn based on Tables A1, A2 in Supplementary Material. We again emphasize that μ_Δ_1__ represents a scientifically relevant effect, which is a constant parameter in the ABT method. We do not study the influence of misspecification in this data example, but merely examine the influence of the variation in the definition of a scientifically relevant ES.

## 4. Discussion

Defining fROIs remains a troublesome issue in fMRI research. Recent studies (e.g., Smith et al., 2011) stress the problematic consequences of inaccurately localizing these fROIs, leading to a loss in validity of subsequent analyses. Typically, thresholding classical *p*-values in an SPM is varied to increase spatial accuracy and consistency in defining these regions of interest. However, not only FPs but also FNs have a detrimental effect on the definition of an fROI. It is therefore important to provide guidelines that do not involve *ad hoc* adjustment of thresholding and that do take into account FNs. Nuzzo (2014) explains for a more general testing context how the misuse of NHST may lead to p-hacking in a more general testing context. With the motivation to avoid *ad hoc* adjustments that are often made due to inter-subject variability, we evaluated the alternative-based thresholding (ABT) method of Durnez et al. (2013) in the context of defining fROIs and compared its performance with NHST.

Simulations were used to evaluate the information captured in the different layers in the LSPM. First, it was shown that relevant information was preserved in the active layer, which is a subset of the layer with voxels detected by NHST, while controlling the FP rate and excluding scientifically irrelevant voxels. Secondly, results indicated that the inactive layer truly consists of voxels that are either noise voxels or scientifically irrelevant, which we can safely exclude as part of the fROI. Third, the uncertainty layer, the added information above and beyond NHST, contains a substantial amount of the scientifically relevant voxels in multiple parameter configurations, while the practically insignificant layer, or the information that is also labeled as part of the fROI with NHST, does not. These findings were corroborated in the real data example. Additionally, this example also showed that thresholding alternative *p*_1_-values makes it possible to increase the specificity of the active layer while maintaining a high sensitivity as evaluated by a benchmark of scientifically relevant effect sizes. If there is some loss in sensitivity, however, valuable information for the definition of the fROI can again be found in the uncertainty layer, while the practically insignificant layer mainly consists of irrelevant information. Combined, these results show that the ABT method is very flexible and that it can be used in a way that is completely suitable for any given context. In any case, the active layer provides a valid definition of a certain region by balancing FPs and FNs through direct control of both types of errors. Depending on the context, one can choose to include the uncertainty layer, especially when any FN can have detrimental consequences.

In order to use the ABT procedure described here, one has to define an (unstandardized) effect size that is deemed scientifically relevant in the context of the fROI and its variance. Such relevant effect sizes have to be defined for power analyses as well. Desmond and Glover (2002) demonstrate how to estimate distributions of different signal components, namely % BOLD signal change (i.e., unstandardized effect size), within- and between-subject variability within ROIs. Mumford and Nichols (2008) provide a more flexible method for power calculation. They extend the work of Desmond and Glover (2002) by making it more flexible through the improvement of the estimation of the within-subject variability and the addition of temporal autocorrelation for multiple designs. Hence, using their methods for estimation, effect sizes could be defined using data from previous research, both personally obtained pilot data or freely available data. When such effect sizes are measured on a different scale than the data at hand, the different signal components can be used to transform these effect sizes.

The data to base effect size estimation on should be independent from the localizer data that is used to define the fROI to avoid circularity of the results. Additionally, we would recommend using high-quality large-scale open-source projects data such as the Human Connectome Project data (Van Essen et al., 2012) to obtain the least biased estimates for effect sizes. For functional localization, defining effect sizes based on prior estimates becomes even more complex due to the possibly high inter-subject variability. One typically aims to define subject-specific fROIs. A practical advice for functional localization to account for this inter-subject variability could be to add an additional run in each subject solely for the purpose of effect size estimation. These data cannot be included in the test for defining the fROI. This further has the huge advantage that problems with different scaling are avoided. Effect sizes estimated based on the pilot data will be on the exact same scale as those of the experimental data, given that the experimental design and software remain unchanged.

Recently, in the neuroimaging literature, reporting effect estimates is highly recommended (Chen et al., 2016), making it easier to define a relevant effect size. Estimating effect sizes on available data however remains complex. Similar to the voodoo correlations (Vul et al., 2009; Vul and Pashler, 2012), thresholding data in order to estimate a scientifically relevant effect size is circular. As a result, the estimated scientifically relevant effect size will be too high, leading to the loss of relevant information using the ABT method. In the context of functional localization, we advise to estimate effect sizes using anatomical masks of the ROI that has to be defined functionally, without relying on thresholding. Recently, substantial improvements in brain atlases have been made. Atlases are no longer purely anatomical, but both functional and spatial properties help shape the parcellation of the brain (Van Essen et al., 2012; Turner and Geyer, 2014; Glasser et al., 2016). Furthermore, these atlases have the advantage of being validated on large populations.

Specification of the alternative distribution and its mean should be based on information of scientific relevance. From this point of view, misspecification through biased effect size estimation is not an issue, since the researcher only wants to find voxels that have an effect size that is larger than the effect size that he/she deems scientifically relevant in that particular context independent of what the true effect size of activation in that region is. However, if a researcher is very uncertain about the effect size estimate, this uncertainty can be translated in a larger τ value for the alternative distribution, which increases its width.

The main focus in this paper was to evaluate ABT with respect to information contained in different layers when judging evidence against both the null and the alternative and to indicate how this complements NHST. At this point, no optimal decision criterion to create a binary decision (activation—no activation) was developed. Very recently, Kang et al. (2015) have developed a new approach for simultaneous control of error rates in fMRI data analysis. They start from the premise that, similarly to the FN rate, the FP rate should converge to zero in large samples. In their likelihood approach, they obtain both FP and FN rate control by contrasting the null and the alternative to judge evidence. In contrast to our approach, the choice of the alternative is data-driven as it is guided by the data that is analyzed. In future work, we aim to include both *p*_0_ and *p*_1_ into one single test criterion that weighs both types of error that have to be controlled. For this, we could work along the lines of the likelihood paradigm of Kang et al. (2015) and pre- and post-experimental rejection ratios (Bayarri et al., 2016).

We presented the ABT method for one-sided testing of positive activation. In the original study of Gonzalez-Castillo et al. (2012), the authors reported whole-brain activation during the task with both positive and negative brain responses. The method described above can be easily adapted to detect scientifically relevant deactivation during the task by specifying a negative value for μ_Δ_1__ by locating the alternative distribution to the left of the null distribution. *p*_0_ is then a left-sided *p*-value under the null in the direction of the alternative while *p*_1_ is a right-sided *p*-value under the alternative in the direction of the null; the interpretation of both *p*-values remains the same.

Although we evaluated the ABT method in the context of spatially accurate definition of fROIs, the scope of the method is much broader than this area. In pre-surgical planning for example, both control of FPs and FNs is an important issue in the guidance of brain surgery. Furthermore, controlling the FP rate as well as the FN rate has advantages for all cognitive neuroscience studies. Related to this, Gross and Binder (2014) also examined alternative thresholding methods for fMRI data, more specifically an amplitude-based thresholding method that focuses on mean differences in signal amplitude between task conditions in order to localize task-related activity. Although some resemblances can be found between their method and ABT and their underlying motivation, the amplitude-based thresholding method is not able to control both FPs and FNs, while we showed the ability of the ABT to provide direct control of both error types using simulations.

In this paper, voxelwise inference was chosen to define the fROI instead of relying on topological features such as peaks (Chumbley and Friston, 2009; Chumbley et al., 2010). We preferred voxelwise inference since topological features such as peaks are less stable both in number and spatial location (Roels et al., 2015), which makes them less than optimal to define spatially accurate fROIs. Another reason for choosing voxelwise inference is that we focused on the extent of the fROI to be defined. Typically once the fROI is defined, its behavior in the primary task of interest is examined by summarizing the signal of the fROI across the voxels it consists of. Including as much informative voxels as possible is of great importance in the context of this procedure. Simply relying on peak voxels as a summary for an fROI may lead to the exclusion of valuable information and to potential bias of the results of the primary experimental task.

In this study, we used the ABT method as a univariate data analysis method. However, multivariate techniques have proven useful in spatial mapping (Kriegeskorte et al., 2006; Nichols, 2012) and are promising for defining fROIs (Duncan and Devlin, 2011). Independent Component Analysis (ICA; McKeown et al., 1997), for example, decomposes the data into different components combining signals over all voxels and enables to distinguish between task-related signals and artifacts. Spatial activation is derived by relating voxels to components that encompass task-related signal. Hence, this voxelwise testing can greatly benefit from directly controlling both FPs and FNs. Applying ABT when using ICA to define fROIs as developed by Durnez et al. (2013) is an avenue for further research.

A Bayesian framework can provide an alternative for the ABT method that incorporates a scientific relevant effect size in conventional frequentist NHST. In Bayesian methods, researchers first specify a prior distribution, *P*(θ), representing their strength of belief for a range of alternatives. After data collection, the prior distribution is then updated using Bayes rule, resulting in the posterior distribution or the beliefs for the alternative conditioned on the observed data, *P*(θ|*data*). For a more elaborate summary of the basics of Bayesian data analysis, see for example Dienes (2014), Rouder et al. (2016), and Wagenmakers (2007). ABT provides a simple and intuitive counterpart for the popular classical *p*-values that can directly complement many existing testing strategies. Importantly, though Bayesian methods may inherently provide more possibilities to incorporate an alternative, not specifying an alternative can in both conventional NHST and the Bayesian framework lead to rejections of the null hypothesis with scant evidence (Rouder et al., 2016). Bayesian hypothesis testing directly compares evidence in favor of the alternative with evidence in favor of the null hypothesis by using for example a Bayes factor. Future research could explore similar comparisons between evidence against the null hypothesis and evidence against the alternative hypothesis in ABT, respectively the *p*_0_-value and the *p*_1_-value.

Finally, we would like to emphasize that ABT still encompasses NHST. Controlling FPs is still a very important part of the method. However, direct control of the FN rate is provided as well, leading to multiple layers of evidence that can be combined. We acknowledge that *ad-hoc* selection of μ_Δ_1__ and τ remains possible. However, as also pointed out by Rouder et al. (2016), it is important to stress that including assumptions on the alternative to test against, renders valid testing. As NHST does not incorporate this, results cannot be expected to implicate information on effect sizes that are of scientific interest. ABT makes progress in this respect by incorporating evidence against the alternative through the *p*_1_-value (Moerkerke et al., 2006; Durnez et al., 2013). Furthermore, by requiring to specify a functionally relevant effect size before the analysis, researchers will need to write down the arguments for their specific choice, which will then be peer reviewed (similarly to power calculations). This increases transparency of the analysis. Besides the specification of μ_Δ_1__ and τ, both control of the false positive rate and false negative rate still require thresholding levels, α and β. As is the case for any procedure, user-specific choices need to be carefully defined but misuse is always possible. Good research practices require specification of such parameters before the actual analysis.

## Author contributions

JDe carried out the study. RS, JDu, and BM helped designing the study, supervised and read the manuscript thoroughly. JG and PB provided the data set on which the method was evaluated, assisted in writing down the specifics on this and read the manuscript thoroughly.

## Funding

This research was possible thanks to the support of the National Institute of Mental Health Intramural Research Program. This study is part of NIH clinical protocol number NCT00001360, protocol ID 93- M-0170. This research was supported by the Fund for Scientific Research-Flanders (FWO-V), as JDe holds a Ph.D. Fellowship of the Research Foundation—Flanders. RS and BM would like to acknowledge the Research Foundation Flanders (FWO) for financial support (Grant G.0149.14). JDu has received funding from the European Union's Horizon 2020 research and innovation programme under the Marie Sklodowska-Curie grant agreement No 706561.

### Conflict of interest statement

The authors declare that the research was conducted in the absence of any commercial or financial relationships that could be construed as a potential conflict of interest.

## Footnotes

^1^www.fmrib.ox.ac.uk/fsl.
