# Supplementary material for: Introducing Alternative-Based Thresholding for Defining Functional Regions of Interest in fMRI
Source: Front Neurosci. 2017 Apr 21;11:222. doi: 10.3389/fnins.2017.00222 (PMC5399022; doi:10.3389/fnins.2017.00222)
Supplement: Supplementary file 1 [file DataSheet1.pdf]

			Active			Inactive			Uncertainty			Practically Insignificant		
μ_{Δ_1}	α	β	Mean	Median	SD	Mean	Median	SD	Mean	Median	SD	Mean	Median	SD
0.25	0.05	0.1	0.131	0.042	0.239	-0.026	-0.017	0.049	0.002	0.0003	0.029	NA	NA	NA
		0.2	0.206	0.099	0.286	-0.007	-0.003	0.035	0.032	0.02	0.087	0.018	0.014	0.022
		0.3	0.327	0.223	0.334	-0.007	-0.003	0.036	0.059	0.036	0.124	0.03	0.021	0.039
	0.001	0.1	0.316	0.204	0.339	-0.026	-0.017	0.049	0.006	0.001	0.037	NA	NA	NA
		0.2	0.339	0.23	0.3437	-0.006	-0.002	0.035	0.056	0.038	0.092	0.022	0.021	0.02
		0.3	0.403	0.299	0.354	-0.004	-0.002	0.037	0.107	0.081	0.135	0.049	0.039	0.044
0.50	0.05	0.1	0.443	0.349	0.359	-0.006	-0.003	0.037	0.069	0.044	0.138	0.043	0.026	0.058
		0.2	0.552	0.461	0.374	-0.006	-0.003	0.037	0.067	0.056	0.129	0.055	0.029	0.078
		0.3	0.625	0.543	0.379	-0.006	-0.003	0.037	0.049	0.05	0.089	0.063	0.03	0.09
	0.001	0.1	0.489	0.387	0.363	-0.003	-0.002	0.039	0.15	0.118	0.174	0.084	0.065	0.074
		0.2	0.577	0.484	0.373	-0.003	-0.001	0.04	0.212	0.177	0.209	0.117	0.091	0.10
		0.3	0.639	0.553	0.378	-0.003	-0.001	0.041	0.253	0.21	0.229	0.138	0.107	0.119
0.75	0.05	0.1	0.697	0.628	0.386	-0.006	-0.003	0.037	0.043	0.045	0.087	0.07	0.033	0.103
		0.2	0.788	0.712	0.39	-0.006	-0.003	0.037	0.042	0.036	0.055	0.079	0.035	0.118
		0.3	0.852	0.775	0.391	-0.006	-0.003	0.037	0.049	0.05	0.035	0.0844	0.036	0.128
	0.001	0.1	0.709	0.627	0.383	-0.003	-0.001	0.041	0.262	0.234	0.243	0.159	0.122	0.137
		0.2	0.795	0.717	0.388	-0.003	-0.001	0.042	0.344	0.305	0.276	0.181	0.138	0.157
		0.3	0.855	0.777	0.389	-0.003	-0.001	0.042	0.427	0.413	0.229	0.195	0.148	0.171

. Table A.1: Results for the visual + letter/number discrimination (Gonzalez-Castillo et al., 2012) task analyzed with the ABT method. For all layers, the mean effect size, the median effect size and the standard deviation (SD) of the effect size of the voxels in the ES benchmark that were categorized in the respective layer are displayed. The average of these measures was computed across the 100 cross-validation steps. The NA values are the consequence of no voxels being categorized as practically insignificant in those scenarios.

	Significant			Non-significant		
α	Mean	Median	SD	Mean	Median	SD
0.05	0.131	0.042	0.239	-0.006	-0.003	0.371
0.001	0.316	0.204	0.339	-0.003	-0.002	0.421

. Table A.2: Results for the visual + letter/number discrimination (Gonzalez-Castillo et al., 2012) task analyzed with NHST. For both layers, the mean effect size, the median effect size and the standard deviation (SD) of the effect size of the voxels in the ES benchmark that were categorized in the respective layer are displayed. The average of these measures was computed across the 100 cross-validation steps.
